# Biosynthetic Interrogation of Soil Metagenomes Reveals Metamarin, an Uncommon Cyclomarin Congener with Activity against *Mycobacterium tuberculosis*

**DOI:** 10.1021/acs.jnatprod.0c01104

**Published:** 2021-02-23

**Authors:** Lei Li, Logan W. MacIntyre, Thahmina Ali, Riccardo Russo, Bimal Koirala, Yozen Hernandez, Sean F. Brady

**Affiliations:** † Laboratory of Genetically Encoded Small Molecules, The Rockefeller University, 1230 York Avenue, New York, New York 10065, United States; ‡ Rutgers, The State University of New Jersey, International Center for Public Health, 225 Warren Street, Newark, New Jersey 07103, United States

## Abstract

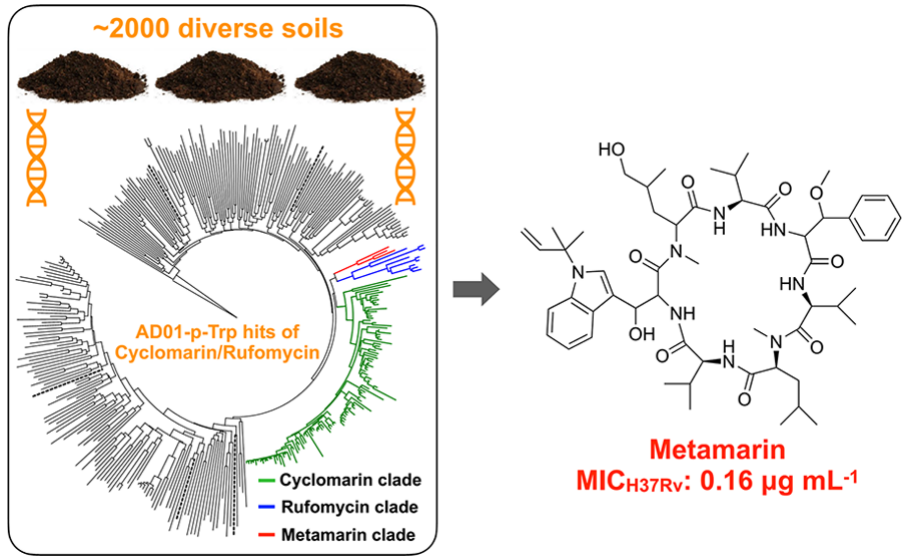

Tuberculosis (TB) remains one of the deadliest infectious diseases. Unfortunately, the development of antibiotic resistance threatens our current therapeutic arsenal, which has necessitated the discovery and development of novel antibiotics against drug-resistant *Mycobacterium tuberculosis* (*Mtb*). Cyclomarin A and rufomycin I are structurally related cyclic heptapeptides assembled by nonribosomal peptide synthetases (NRPSs), which show potent anti-*Mtb* activity with a new cellular target, the caseinolytic protein ClpC1. An NRPS adenylation domain survey using DNA extracted from ∼2000 ecologically diverse soils found low cyclomarin/rufomycin biosynthetic diversity. In this survey, a family of cyclomarin/rufomycin-like biosynthetic gene clusters (BGC) that encode metamarin, an uncommon cyclomarin congener with potent activity against both *Mtb* H37Rv and multidrug-resistant *Mtb* clinical isolates was identified. Metamarin effectively inhibits *Mtb* growth in murine macrophages and increases the activities of ClpC1 ATPase and the associated ClpC1/P1/P2 protease complex, thus causing cell death by uncontrolled protein degradation.

Tuberculosis (TB) remains a major public health threat and is recognized by the World Health Organization (WHO) as the leading infectious disease killer worldwide.^1^ The continued emergence of multidrug-resistant and extensively drug-resistant *Mycobacterium tuberculosis* (*Mtb*) has made the prevention and treatment of TB very challenging.^2^ The discovery and development of anti-*Mtb* drugs with new cellular targets is therefore a high priority. Cyclomarin A and rufomycin I (ilamycin C_1_) are chemically similar cyclic heptapeptide antibiotics^3−6^ (Figure 1a) that are highly potent (nanomolar MIC) against multidrug-resistant *Mtb* as well as other pathogenic nontuberculosis mycobacteria.^7,8^ They have been of particular interest for the development of TB therapeutics as they have a novel mode of action by targeting cellular proteostasis via the protease regulatory chaperone ATPase (ClpC1).^7−10^ The biosynthesis of the cyclic peptide scaffolds for cyclomarin A and rufomycin I follow the colinear extension model of modular NRPS systems.^11−13^ The biosynthesis of cyclomarin A involves a heptamodular NRPS that directly incorporates the nonproteinogenic amino acids *N*-(1,1-dimethyl-1-allyl)-Trp (prenylated-Trp, p-Trp) and 3-amino-3,5-dimethyl-4-hexenoic acid (ADH) into the growing peptide. In contrast the β-hydroxy, δ-hydroxy and β-methoxy substituents seen on p-Trp_1_, Leu_2_ and Phe_4_ (respectively) are thought to be installed while the proteinogenic substrates are tethered to their PCPs.^11^ In rufomycin, the heptamodular NRPS uses three nonproteinogenic amino acids p-Trp, 3-nitro-l-Tyr and L-2-amino-4-hexenoic acid (AHA). The l-Leu residue incorporated at the seventh position of the peptide undergoes post-NRPS cyclization with the amide of neighboring l-Leu to generate the 6-hydroxy-5-methyl-3-amino-2-piperidinone moiety.^12,13^ It should be noted that while ADH of cyclomarin A and AHA of rufomycin I bear a structural resemblance to one another and occur at analogous positions in the two peptides, they represent convergent biosyntheses involving homologation of a valine-derived isobutyraldehyde with pyruvate (cyclomarin A) and a trimodular polyketide synthase assembly line (rufomycin I).^11−13^

**Figure 1:**
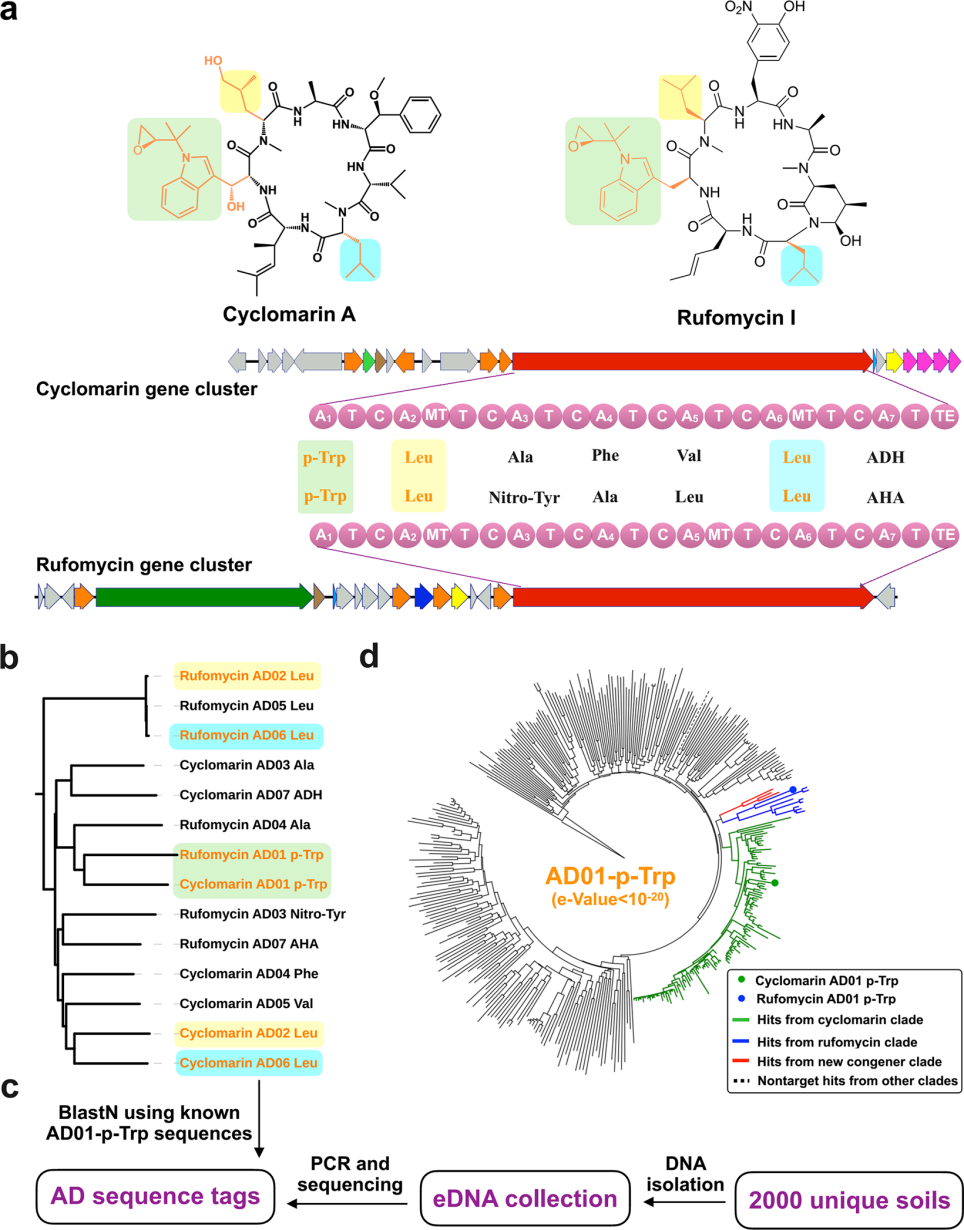
Sequence tag-based screen of cyclomarin/rufomycin-like BGCs. a) The structures and BGCs of cyclomarin A and rufomycin I. b) Phylogenetic tree of all A-domains from cyclomarin A and rufomycin I BGCs. p-Trp, ADH and AHA represent *N*-(1,1-dimethyl-1-allyl)Trp, 3-amino-3,5-dimethyl-4-hexenoic acid and 2-amino-4-hexenoic acid, respectively. c) Pipeline for the discovery of cyclomarin/rufomycin congeners from the soil metagenome. eDNA isolated from ∼2000 unique soils was screened by PCR using universal A-domain degenerate primers. The reads from these sequenced A-domain amplicons were analyzed by BlastN using the most conserved AD01-p-Trp reference sequences. d) Phylogenetic tree of AD01-p-Trp domains from the two reference BGCs and AD01-p-Trp-like BlastN-processed A-domains from screened soil metagenomes.

To expand our search for cyclomarin/rufomycin-like antibiotics, here we focus on soil metagenomes. Due to the complexity of an individual soil microbiome, it is challenging to sequence soil metagenomes to a depth that permits the discovery of rare natural product BGCs.^14−16^ We have developed a culture-independent BGC discovery strategy that uses degenerate PCR primers targeting conserved biosynthetic genes to explore secondary metabolite diversity in complex metagenomes.^17−19^ In this method, sequenced PCR amplicons (Natural Product Sequence Tags, NPSTs) derived from either metagenomic libraries or DNA extracted directly from environmental samples are aligned to a reference collection of domain sequences from characterized metabolites to identify BGCs of interest.^17−19^ In this phylogenetic analysis, amplicons that cluster together with domain sequences from BGCs of interest are used to guide the recovery of new BGCs from metagenomic libraries. Natural products are then accessed from metagenome-derived BGCs by heterologous expression. In this study, NRPS A-domain sequence tags from ∼2000 ecologically and geographically diverse soils were used to evaluate cyclomarin/rufomycin-family biosynthetic diversity in the soil microbiome. This information was used to guide the search for other cyclomarin/rufomycin-like structures, resulting in the discovery of metamarin, a novel anti-*Mtb* compound that may represent a simplified evolutionary precursor to cyclomarin A.

## Results and Discussion

### Metagenomic Survey of Cyclomarin/Rufomycin-Family Biosynthetic Diversity

In the biosynthesis of cyclomarin A and rufomycin I, the same amino acids are incorporated at three positions of their macrocyclic peptide scaffolds. These include the use of p-Trp by the first A-domain, and Leu by the third and sixth A-domains (Figure 1a).^11−13^ A phylogenetic analysis of all cyclomarin/rufomycin A-domain sequences indicated that the domains responsible for incorporating p-Trp are most highly conserved among these two evolutionarily related BGCs (Figure 1b) and thus we focused on this domain to track cyclomarin/rufomycin-like BGCs in NPST data from soil metagenomes. DNA extracted from ∼2,000 soils was used as template in PCR reactions with A-domain-specific degenerate primers (Figure 1c and Supporting Information (SI) Table S1). The resulting amplicons were sequenced and soil A-domain NPSTs were compared by BlastN to the cyclomarin A and rufomycin I p-Trp A-domain sequences. An A-domain phylogenetic tree (Figure 1d) derived from the sequence tags that are most closely related to these A-domains contains three closely related clades that we predicted were derived from cyclomarin/rufomycin congener BGCs. The largest group of sequences falls into a clade that contains the cyclomarin A p-Trp A-domain and a second smaller clade contains the rufomycin I A-domain sequences. The third smaller clade contains no known p-Trp A-domain sequences, which suggested to us that it might arise from BGCs that encode a novel cyclomarin/rufomycin congener. To identify the potential new congener encoded by BGCs associated with this new clade, and further explore the existing cyclomarin/rufomycin clades, we turned our sequencing efforts to a collection of archived metagenomic libraries from which target BGCs can be readily recovered and the metabolites they produce can be accessed by heterologous expression.

### Cyclomarin/rufomycin-Like BGCs from Metagenomic Cosmid Libraries

**Figure 2:**
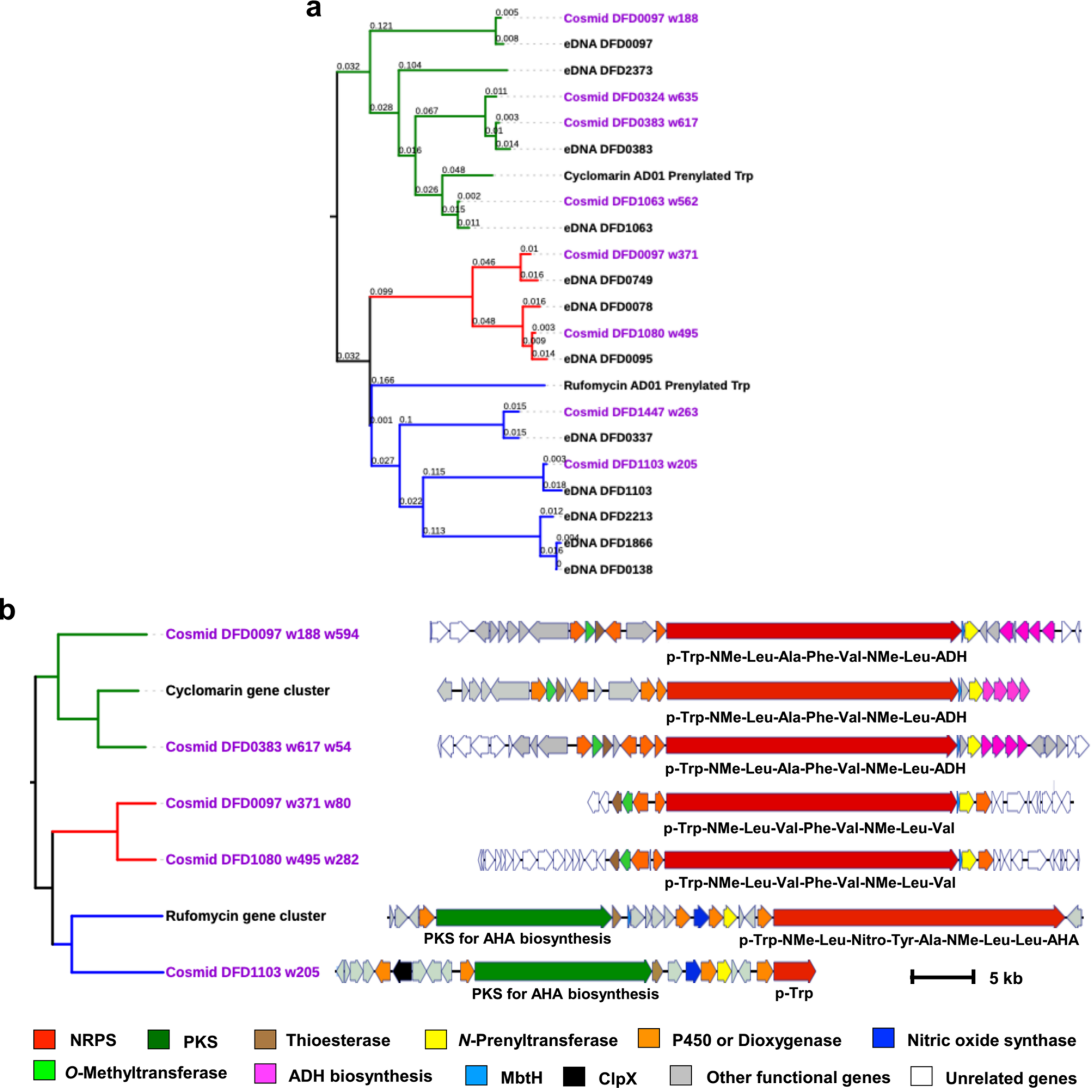
Positioning and analysis of cyclomarin/rufomycin-like BGCs from archived cosmid libraries. (a) Mapping cyclomarin/rufomycin-like hits from eDNA collection to archived cosmid libraries. (b) Summary of all cyclomarin/rufomycin-like BGCs recovered from archived cosmid libraries.

As part of our ongoing soil metagenomic-guided natural product discovery program, we have constructed a series of saturated cosmid libraries to use for recovering BGCs of interest.^15,16^ Each library contains more than 20-million unique cosmid clones that are arrayed in sets of 384 subpools containing on average ∼25 000 unique clones. Purified cosmid DNA from each pool was screened with the same A-domain degenerate primers that were used to screen soil DNA. BlastN analysis of this collection of library-derived A-domain amplicon sequences identified eight cyclomarin/rufomycin-like p-Trp NPSTs from six different eDNA libraries. These NPSTs span all three subclades we identified in the original soil screen, suggesting that the BGCs captured in our archived metagenomic libraries are representative of the cyclomarin/rufomycin-like biosynthetic diversity that we identified in ∼2000 soil metagenomes (Figure 2a). Using the specific metagenomic libraries from which these sequences were amplified, we recovered collections of overlapping cosmid clones associated with two amplicons from the cyclomarin A clade, one from the rufamycin clade, and two from the novel clade. Each was sequenced, assembled, and annotated to reveal a cyclomarin/rufomycin-like BGC (Figure 2b).

As suggested by our NPST analysis, two recovered BGCs (DFD0097_w188_w594 and DFD0383_w617_w54) are predicted to encode cyclomarin. This prediction is based on an A-domain substrate specificity analysis and the collection of biosynthetic genes present in each BGC (Figure 2b). Although the entire BGC associated with the rufamycin-like NPST was not recovered from the metagenomic library, the portion we did recover (UT60_w205) closely resembles the rufomycin BGC. Most of the proteins encoded by UT60_w205 show high sequence identity (46–70%) to proteins found in the rufomycin BGC (SI Figure S1). In addition, the substrate specificity prediction for the first A-domain together with the collection biosynthetic enzymes encoded on this clone suggest that this eDNA BGC produces all three of the rare building blocks found in rufomycin: p-Trp, 3-nitro-Tyr and 2-amino-4-hexenoic acid (AHA) (SI Figure S1). Interestingly, this BGC is predicted to encode a ClpX-like ATPase.^20,21^ Considering that rufomycin targets the ClpC1 ATPase,^7,10^*clpX* might function as a self-resistance gene.^22,23^ The two BGCs recovered (DFD0097_w371_w80 and DFD1080_w495_w282), that are associated with NPSTs from the clade without any previously known sequences, are 90% identical to each other and predicted to encode identical collections of tailoring enzymes. Based on A-domain amino acid specificity predictions, they are predicted to encode a novel cyclomarin-like heptapeptide with the following sequence: p-Trp_1_-*N*-Me-Leu_2_-Val_3_-Phe_4_-Val_5_-*N*-Me-Leu_6_-Val_7_ (Figure 2b). We have called these the metamarin (metagenomic cyclomarin) or *met* BGCs. Finally, to determine whether other clades in the p-Trp A-domain phylogenetic tree were derived from BGCs that might also encode cyclomarin/rufomycin-like natural products, we recovered and sequenced cosmid clones associated with five additional A-domain NPSTs distributed around the A-domain phylogenetic tree (Figure 1d). All of these BGCs are predicted to encoded NPRS- or hybrid NRPS-PKS-derived structures that are not related to cyclomarin or rufomycin. While we did not exhaustively sample all clades in the p-Trp A-domain phylogenetic tree, this analysis suggests that NPSTs derived from cyclomarin/rufomycin-like BGCs are likely restricted to the clades we have explored in this study. Based on the frequency that we see NPSTs from different A-domain clades, the most common BGCs from this family encode cyclomarin, followed by rufomycin and finally metamarin (Figure 1d). This may explain why metamarin remained undiscovered in previous natural product discovery efforts. Herein, we describe the characterization of a new cyclomarin-like natural product that is encoded by the *met* BGCs.

### Heterologous Expression, Structure Elucidation and Predicted Biosynthesis of Metamarin

**Figure 3:**
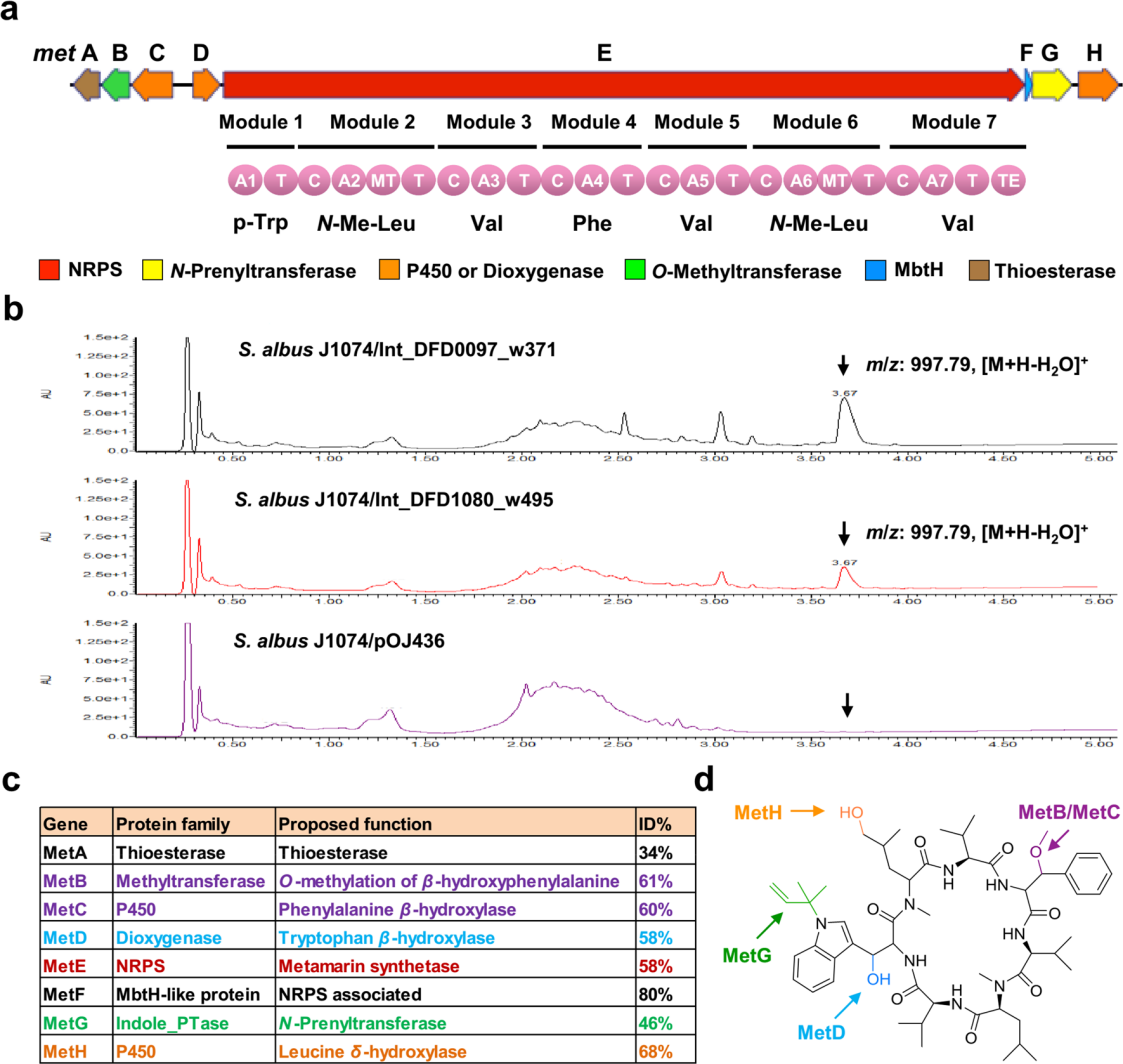
Heterologous expression, structure and predicted biosynthesis of **1**. (a) BGC of **1**. (b) LCMS analysis of culture broth extracts of *S. albus* J1074 harboring Int_DFD0097_w371, Int_DFD1080_w495 or pOJ436. The clone-specific metabolite **1** was monitored at 3.67 min. (c) Predicted functions of proteins encoded by the *met* BGC. ID% represents the amino acid identities of protein homologues encoded by **1** and cyclomarin A BGCs. (d) Chemical structure of **1**.

In silico analysis of cosmids DFD0097_w371 and DFD1080_w495 suggested that both contain entire cyclomarin-like BGCs (Figure 3a). The integration-resistance [ΦC31-*acc(3)IV*] cassette from the plasmid pOJ436 was separately inserted into the two cosmids using traditional molecular cloning methods,^24^ thus generating *Streptomyces* integrative cosmids, Int_DFD0097_w371 and Int_DFD1080_w495 (SI Figure S2). For heterologous expression, the two integrative cosmids and the empty pOJ436 vector were individually introduced into *Streptomyces albus* J1074. The exconjugants were fermented in R5a medium, and mature cultures were extracted using HP20 resin. LC-MS analysis indicated that both cultures produced a BGC specific peak with the same retention time and mass (*m*/*z* 997.79) (Figure 3b), suggesting these two very closely related BGCs (∼90% nucleotide sequence identity for homologous genes) confer the production of the same metabolite to *S. albus.* As shown in Figure 3b, *S. albus* harboring Int_DFD0097_w371 had a higher titer compared to that harboring Int_DFD1080_w495. The metamarin BGCs in these clones have the same gene organization and show ∼90% overall sequence identity. These small changes in sequence likely cause the observed difference in titer. Culture broth extracts from *S. albus* harboring Int_DFD0097_w371 were used to purify the clone-specific metabolite (23.3 mg/L), which we have named metamarin (**1**).

The structure of **1** was determined using a combination of high-resolution MS and 1D/2D NMR data (SI Figures S3–12 and Table 1). The molecular formula of **1** was determined to be C_55_H_82_N_8_O_10_ by high-resolution electrospray ionization mass spectrometry (HRESIMS) from *m*/*z* 997.6102 [M+H–H_2_O]^+^ (calcd for C_55_H_81_N_8_O_9_^+^, 997.6121) (SI Figure S3a). The ^1^H–^15^N HSQC NMR spectrum contained only five cross-peaks, immediately indicating the presence of five proton-attached ^15^N atoms (δ_H_ 7.11, 7.20, 8.09, 8.20, and 8.60) with δ_H_ values characteristic of five amide groups/functionalities. The ^1^H NMR spectrum contained two singlet methyl resonances (δ_H_ 2.74 and 2.82), consistent with *N*-methyl substituents. The ^1^H–^15^N HMBC NMR spectrum confirmed that these signals correlate to two distinct ^15^N atoms that bear no protons. These data indicated the presence of seven amino acid residues, which was corroborated by seven distinct cross peaks in the ^1^H–^13^C HSQC NMR spectrum with δ_H_ and δ_C_ values diagnostic of the amino acid α-position (4.03–4.88 and 54.5–60.3, respectively) and by seven carbonyl resonances in the ^13^C NMR spectrum (δ_C_ 168.7–172.5). The structure of each amino acid side chain was determined using COSY, ^1^H–^13^C HSQC and ^1^H–^13^C HMBC NMR spectra. Of note, the *N*-1,1-dimethyl-1-allyl substituent was placed using a ^1^H–^13^C HMBC NMR correlation from H-4 to C-12, and also using ^1^H–^15^N HMBC NMR correlations from H-4, H-13, H-15, and H-16 to N-1’. A hydroxy substituent was placed at the β-position of this same residue on the basis of chemical shifts at this position (δ_H_ 5.33 and δ_C_ 69.3). The connectivity of these seven partial structures was established using HMBC correlations from the nitrogen-attached amide protons (or *N*-methyls) of each residue to the carbonyl of its *N*-terminal neighbor.

**Table 1:** ^1^H (600 MHz) and ^13^C NMR (150 MHz) data of 1 in CDCl_3_

position		δ_C_, Type		δ_H_ (J in Hz)	COSY	^1^H–^13^C HMBC	^1^H–^15^N HMBC
*N*-(1,1-dimethyl-1-allyl)-β–OH-Trp	1	171.4	C				
	2	54.5	CH	4.63, m	3, 8’	1, 3, 5	8’
	3	69.3	CH	5.33, d(4.9)	2	1, 2, 4, 5, 6	8’
	4	123.4	CH	7.32, s		2, 3, 5, 6, 11, 1	1’
	5	111.5	C				
	6	127.0	C				
	7	119.3	CH	7.56, d(7.6)	8	5, 6, 9, 11	
	8	119.6	CH	7.06, t(7.3)	7	6, 7	
	9	121.7	CH	7.13, t(7.7)	10	7, 8, 10, 11	
	10	114.5	CH	7.51, d(8.4)	9	6, 8	1’
	11	135.9	C				
	12	59.3	C				
	13	143.8	CH	6.08, dd(17.6, 10.7)	14	12, 15, 16	1’
	14a	114.0	CH_2_	5.18, d(17.8)	13	12, 13, 15, 16	1’
	14b			5.23, d(10.8)	13	12, 13, 15, 16	1’
	15	27.9a	CH_3_	1.72, s		12, 13, 16	1’
	16	28.0a	CH_3_	1.73, s		12, 13, 15	1’
	8’			7.20, m	2	2, 3, 17	

Val_1_	17	172.5	C				
	18	59.4	CH	4.03, t(9.7)	19, 7’	17, 19, 20, 21, 22	7’
	19	31.3	CH	0.80, m	18, 20, 21	17, 18, 20, 21	7’
	20	20.0a	CH_3_	0.63, d(6.5)	19	18, 19, 21	
	21	18.8	CH_3_	0.66, d(6.5)	19	18, 19, 20	
	7’			8.20, d(9.2)	18	17, 18, 22	

*N*-Me-Leu_1_	22	168.7	C				
	23	59.0	CH	4.79, dd(10.4, 3.3)	24a, 24b	22, 24, 25, 6’	6’
	24a	38.9	CH_2_	1.13, m	23, 24b, 25	22, 23	6’
	24b			2.26, m	23, 24a, 25	22, 23, 25, 26, 27	6’
	25	25.2	CH	1.49, m	24a, 24b, 26, 27	23, 24, 26, 27	
	26	22.6	CH_3_	0.89, d(6.9)	25	24, 25, 27	
	27	22.6	CH_3_	0.93, t(6.9)	25	24, 25, 26	
	6’	29.7		2.82, s		28, 23	6’

Val_2_	28	170.7	C				
	29	55.3	CH	4.43, t(8.3)	30, 5′	28, 30, 31, 32	5′
	30	31.1	CH	2.22, m	29, 31, 32	28, 29, 31, 32	5′
	31	20.1a	CH_3_	0.97, d(6.6)	30	29, 30, 32	
	32	19.3	CH_3_	1.09 d,(6.6)	30	29, 30, 31	
	5′			8.09, d(7.2)	29	29, 30, 33	

β-OMe-Phe	33	170.0	C				
	34	56.7	CH	4.88, t(4.8)	35, 4’	33, 35, 36, 43	4’
	35	80.5	CH	5.10, d(5.3)	34	33, 34, 36, 37–41, 42	4’
	36	135.2	C				
	37–41	127.0–128.8	CH	7.19–7.25, m		35, 36, 37–41	
	42	57.8	CH_3_	3.34, s		35	
	4’			7.11, d(4.6)	34	33, 34, 35, 43	

Val_3_	43	171.0	C				
	44	60.3	CH	4.63, m	45, 3′	43, 45, 46, 47, 48	3′
	45	32.0	CH	1.93, m	44, 46, 47	43, 44, 46, 47	3′
	46	18.1	CH_3_	0.73, d(6.9)	45	44, 45, 47	
	47	20.2a	CH_3_	0.93, t(6.9)	45	44, 45, 46	
	3′			8.60, d(10.4)	44	44, 48	

*N*-Me-δ−OH-Leu_2_	48	169.4	C				
	49	59.6	CH	4.74, d(11.1)	50a, 50b	1, 48, 50, 51, 2’	2’
	50a	32.7	CH_2_	0.33, m	49, 50b, 51	48, 49, 51, 52, 53	2’
	50b			2.20, m	49, 50a, 51	48, 49, 51, 52, 53	2’
	51	33.5	CH	1.40, m	50a, 50b, 52, 53	49, 50, 52, 53	
	52a	66.4	CH_2_	3.14, m	51, 52b	50, 51, 53	
	52b			3.18, m	51, 52a	50, 51, 53	
	53	17.7	CH_3_	0.66, d(6.5)	51	50, 51	
	2’	30.1		2.74, s		1, 49	2’

a Overlapping signals may be interchanged.

Marfey’s method was employed for configurational assignment of the three Val residues and one *N*-Me-Leu residue in **1**.^25^ Based on comparison of retention times of FDAA-derivatized standard amino acids to FDAA-derivatized amino acids in the hydrolysate of **1**, all Val residues as well as the *N*-Me-Leu residue were determined to be L-configured in **1**. Compound **1** differs from cyclomarin A at three amino acid positions. The p-Trp at position one contains a double bond in **1** instead of an epoxide. The alanine and the 3-amino-3,5-dimethyl-4-hexenoic acid (ADH) at positions 3 and 7, respectively are both valines in **1** (Figure 3). The cyclomarin congener M10709, which is produced by *Streptomyces* sp. IFM 10709 shares a similar structure to that of **1**.^26^ Where **1** contains a valine at the third residue M10709 contains an alanine (SI Figure S13). Unfortunately, neither the anti-*Mtb* activity nor the BGC for this congener has been reported.^25^ As the M10709 BGC has not been sequenced we do not know where its p-Trp A-domain falls in the A-domain phylogenetic tree (Figure 1d).

Based on our final structure, the biosynthesis of **1** is expected to follow the colinear extension model of modular NRPS systems, starting with AD01-p-Trp and ending with AD07-Val (SI Figure S14). Subsequent peptide release and macrocyclization are predicted to occur via the C-terminal thioesterase (TE) domain of MetE. Furthermore, the methyltransferase (MT) domains in the second and sixth modules of MetE are predicted to carry out the observed *N*-methylation of AD02-Leu and AD06-Leu, respectively (SI Figure S14). In a BlastP search, most proteins encoded by the BGC of **1** returned top hits that corresponded to homologues encoded by the cyclomarin A BGC from *Salinispora arenicola* CNS-205 (Figure 3c). The *N*-prenyltransferase, MetG, is predicted to be responsible for *N*-prenylation of the tryptophan residue, thus generating the unique, nonproteinogenic p-Trp building block (SI Figure S14). Based on high sequence similarity to enzymes from cyclomarin A BGC, the cytochrome P450, and methyltransferase, MetC and MetB, are predicted to be jointly involved in the β-oxidation/methylation of AD04-Phe. Two additional oxidative enzymes, MetD and MetH, are predicted to be involved in the β-hydroxylation of AD01-p-Trp and δ-hydroxylation of AD02-Leu, respectively (Figure 3c). The absence of an epoxide in the structure of **1** is supported by the fact that the BGC of **1** does not contain a close relative of the cytochrome P450 that is responsible for introducing the p-Trp epoxide in cyclomarin. It is interesting to note that this gene is also missing from one of the cyclomarin-like eDNA derived BGCs (DFD0097_w188_w594) suggesting that it may actually encode the production of an *N*-(1,1-dimethyl-1-allyl)-Trp version of cyclomarin.^11,27^

### Antimicrobial Activity and Mode of Action of 1

Compound **1** has a narrow spectrum of activity. Among the strains tested, **1** is selectively active against mycobacteria and *Micrococcus luteus*. Compound **1** has an MIC of 8 and 16 μg mL^–1^ against *M. luteus* and *Mycobacterium smegmatis*, respectively (SI Table S2). Furthermore, **1** exhibited potent activity against *Mtb* H37Rv with an MIC of 0.16 μg mL^–1^ (SI Table S2). Most notably, **1** also exhibited potent activities against three different multidrug-resistant *Mtb* clinical isolates with MICs of 0.08–0.63 μg mL^–1^, which is comparable to that of cyclomarin A (SI Table S2).

**Figure 4:**
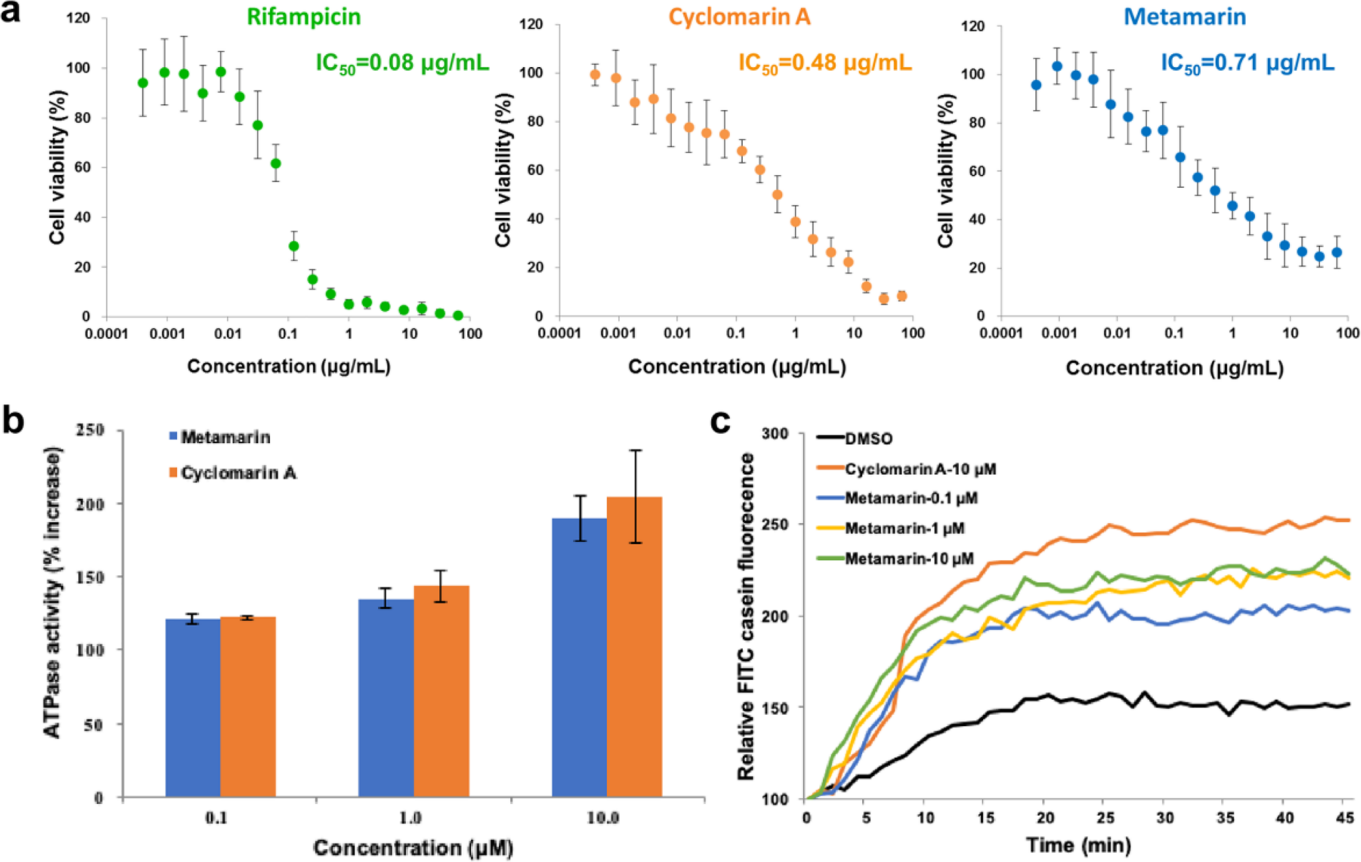
Intracellular anti-*Mtb* activity and mode of action of **1**. (a) Cell viability of *Mtb*-infected murine macrophages in response to treatment of **1**. Cell viability was observed based on intercellular luminescence measurement. Rifampicin and cyclomarin A were used as the controls. (b) ClpC1 ATPase activity and c) proteolytic activity of ClpC1/P1/P2 complex in response to treatment of **1**. Initial FITC-casein fluorescence was set as 100 and relative changes in fluorescence were recorded. Cyclomarin A was used as the positive control. These experiments were carried out in triplicate.

*Mtb* is capable of surviving and replicating in macrophages, which normally play a central role in recognizing and destroying invading pathogens.^28^ Cyclomarin A has been shown to kill *Mtb* in both mouse bone marrow and THP1 derived macrophages.^29^ We therefore tested **1** for anti-*Mtb* activity in a murine macrophage model. In this model, J774A.1 mouse macrophages infected with *Mtb* harboring the mLux plasmid were treated with **1**, and after 3 days, residual bacterial cell viability inside the macrophages was determined by luminescence measurements. Compound **1** effectively inhibited *Mtb* growth in a concentration-dependent manner with an IC_50_ of 0.71 μg mL^–1^, which is comparable to that of cyclomarin A (Figure 4a). Considering that there is a general correlation between activity in macrophages and mouse models,^30^ it will be interesting to evaluate the in vivo activity of **1**.

As both cyclomarin A and rufomycin I bind the *Mtb* ClpC1 ATPase,^7,9^ we expected that **1** would do the same. To explore the mode of action of **1** against *Mtb*, we used two enzyme assays that were developed to probe different aspects of the ClpC1/P1/P2 protease complex.^7,9^ As shown in Figure 4b, the cyclic peptide **1** significantly stimulated ClpC1 ATPase activity at a concentration of 10 μM. As shown in Figure 4c, **1** also increased the proteolytic activity of the ClpC1/P1/P2 complex. The impact of **1** on ClpC1 ATPase activity and ClpC1/P1/P2 proteolysis mimics that of cyclomarin A (Figure 4b,c).^9^

## Concluding Remarks

Compound **1**, like cyclomarin A, appears to bind ClpC1 and cause cell death by deregulation of the ClpC1/P1/P2 protease complex.^9^ The most significant difference between cyclomarin A and **1** is the change of the seventh amino acid from ADH to valine (Figure 1a and 3d). The ADH moiety in cyclomarin A is encoded by a four-gene cassette that is not present in the *met* BGC.^11^ Interestingly, in rufomycin I this position has a different long hydrophobic nonproteinogenic amino acid (AHA) that is encoded by a distinct PKS cassette (Figure 1a,d).^12,13^ It is not clear from the structure of cyclomarin A bound to *Mtb* ClpC1 what evolutionary advantage the incorporation of these large hydrophobic building blocks would have over valine. However, the selective recruitment of different multigene cassettes to the cyclomarin A and rufomycin I BGCs suggests the switch from a valine to a larger hydrophobic residue may be evolutionarily advantageous. The discovery of three structurally distinct hydrophobic amino acids at this position suggests the optimization of this site may still be an ongoing process in nature and future exploration of this position by chemical synthesis could prove productive.

## Experimental Section

### General Experimental Procedures

All reagents were purchased from commercial sources and used without further purification. All solvents used for chromatography were HPLC grade or higher. Optical rotation was measured using a Jasco P-1020 digital polarimeter (P-103T temperature controller) with a 50 mm microcell (1.2 mL). Infrared (IR) spectra were acquired on a Bruker Optics Tensor 27 FTIR spectrometer using an attenuated total reflection attachment. UV–vis spectra were recorded on a Nandrop ND-1000 spectrophotometer. For all liquid chromatography, solvent A = H_2_O (0.1% v/v formic acid) and solvent B = CH_3_CN (0.1% v/v formic acid). UPLC-LRMS data were acquired on a Waters Acquity system equipped with QDa and PDA detectors, a Phenomenex Synergi Fusion-RP 80 Å column (2.0 × 50 mm, 4 μm) and controlled by Waters MassLynx software. The following chromatographic conditions were used for UPLC-LRMS: 5% B from 0.0 to 0.9 min, 5% to 95% B from 0.9 to 4.5 min, 95% B from 4.5 to 5.0 min, 95% to 5% B from 5.0 to 5.4 min, and 5% B from 5.4 to 6 min (flow rate of 0.6 mL/min and 10 μL injection volume). UPLC-HRMS data were acquired on a SCIEX ExionLC UPLC coupled to an X500R QTOF mass spectrometer, equipped with a Phenomonex Kinetex PS C18 100 Å column (2.1 × 50 mm, 2.6 μm) and controlled by SCIEXOS software. The following chromatographic conditions were used for UPLC-HRMS unless noted otherwise: 5% B from 0.0 to 1.0 min, 5% to 95% B from 1.0 to 10.0 min, 95% B from 10.0 to 12.5 min, 95% to 5% B from 12.5 to 13.5 min, and 5% B from 13.5 to 17.0 min (flow rate of 0.4 mL/min and 1 μL injection volume). The following ESI+ HRMS conditions were used: temperature of 500 °C, spray voltage of 5500 V and collision energy of 10 V. Automated flash column chromatography was performed using a CombiFlash Rf200 system (Teledyne ISCO) equipped with a 100 g Gold HP C18 column and UV/ELSD detection. Semipreparative HPLC was performed on an Agilent 1200 Series HPLC with UV detection and equipped with an XBridge Prep C18 130 Å column (10 × 150 mm, 5 μm). ^1^H, ^13^C, COSY, ^1^H–^13^C HSQC, and ^1^H–^13^C HMBC NMR spectra of **1** were acquired on a Bruker Avance DMX 600 MHz spectrometer (The Rockefeller University, New York, NY). ^1^H–^15^N HSQC and ^1^H–^15^N HMBC NMR spectra were acquired on a Bruker Avance III HD 500 MHz spectrometer (Weill-Cornell Medicine, New York, NY). Both instruments were equipped with cryogenic probes. All spectra were recorded at room temperature in CDCl_3_. Chemical shift values are reported in ppm and referenced to residual solvent signals: 7.26 ppm (^1^H) and 77.16 ppm (^13^C).

### Screening soils for AD01-p-Trp-Like Tags of Cyclomarin/Rufomycin-Family Compounds

eDNA was extracted from soil samples using a previously established protocol.^17−19^ Briefly, 25 g of each soil was heated in lysis buffer (100 mM Tris-HCl, 100 mM EDTA, 1.5 M NaCl, 1% CTAB, 2% SDS, pH 8.0) at 70 °C with gentle mixing for 2 h. Soil particulates were removed from the lysate by centrifugation and 0.6 volumes of isopropanol were added to the resulting supernatant for eDNA precipitation. After centrifugation (12 000 rpm/10 min), the eDNA pellets were washed with 70% ethanol and dried at room temperature for 2 h. Finally, the eDNA pellets were resuspended in 500 μL of TE (10 mM Tris-HCl, 1 mM EDTA, pH 8.0), which were screened with A-domain degenerate primers (SI Table S1). To distinguish PCR amplicons generated from each soil sample, Illumina MiSeq sequencing adapters and a collection of different 8 bp barcodes as well as 1–4 bp spacer sequences were added into the degenerate primers. PCR reaction mixtures (12 μL): 1X FailSafe G Buffer (Lucigen), 0.5 μL of each primer (10 μM), 0.1 μL OmniTaq (DNA Polymerase Technology) and 100 ng eDNA. PCR reaction conditions for A-domain amplification: 95 °C/5 min, (95 °C/30 s, 63.5 °C/30 s, 72 °C/45 s) × 35 cycles, 72 °C/5 min. PCR reaction mixtures for each soil sample were pooled and size-selected for ∼700-bp PCR products by gel electrophoresis. The mixed PCR products were sequenced using a MiSeq Reagent Nano Kit v3 on a MiSeq sequencer (Illumina). The amplicons were demultiplexed into the corresponding soil samples and trimmed to 416 bp of the combined reads (240 bp of the forward read, a single “N” spacer and 175 bp of the reverse-complemented reverse read). Then, the trimmed reads were clustered at 95% identity across the same soil samples, thus generating NPSTs of soil metagenomes. These NPSTs were then searched using BlastN against the two manually curated AD01-p-Trp sequences from cyclomarin A and rufomycin I BGCs. A-domain amplicons that matched cyclomarin A or rufomycin I AD01-p-Trp at an e-value <10^–20^ were considered as hits. A multiple sequence alignment of all qualifying hit sequences was generated using MUSCLE,^31^ and the resulting alignment file was used to generate a maximum-likelihood tree with FastTree.

### Clone Recovery for New Cyclomarin/Rufomycin-Like BGCs

In this study, previously archived soil eDNA cosmid libraries were probed to recover cyclomarin/rufomycin-like BGCs. Construction, PCR screening with barcoded A-domain degenerate primers, amplicon sequencing and read processing for these cosmid libraries have been described in detail previously.^17−19^ Using the eDNA-derived AD01-p-Trp-like hits in the well-defined clade as references, these amplicon sequences were then analyzed by our previously developed bioinformatic platform eSNaPD (environmental Surveyor of Natural Product Diversity) software package,^32^ thus generating a panel of p-Trp-like hits from cosmid libraries. The library well locations for targeted hits were identified by the barcode parsing functionality of the eSNaPD software. Then, specific primers targeting each unique sequence of interest were designed manually (SI Table S1). Single cosmids were recovered from library wells of interest using a serial dilution PCR strategy described previously.^18,19^ The recovered cosmids were sequenced using a MiSeq Reagent Nano Kit v2 on a MiSeq sequencer (Illumina). Then, sequence reads were assembled into contigs using Newbler 2.6 (Roche). The final assembled BGCs were analyzed using antiSMASH 5.0 to predict the amino acid specificity of each A-domain domain.^33^

### Heterologous Expression

The integration-resistance (ΦC31-*acc(3)IV*) cassette was obtained by digesting the plasmid pOJ436 with *Dra* I, and then ligated into the *Psi* I-digested linear cosmids DFD0097_w371 or DFD1080_w495, thus generating the integrative cosmids, Int_DFD0097_w371 and Int_DFD1080_w495, respectively.^24^ For heterologous expression, the two integrative cosmids and the empty pOJ436 vector were individually introduced into *Streptomyces albus* J1074 via intergenic conjugation. Then, the resultant conjugants were used to seed starter cultures in 50 mL trypticase soy broth (TSB) and these cultures were shaken for 36 h (30 °C/200 rpm). 500 μL of each seed culture was transferred into 50 mL R5a production medium [100 g/L sucrose, 10 g/L glucose, 5 g/L yeast extract, 10.12 g/L MgCl_2_·6H_2_O, 0.25 g/L K_2_SO_4_, 0.1 g/L casamino acids, 21 g/L MOPS, 2 g/L NaOH, 40 μg/L ZnCl_2_, 20 μg/L FeCl_3_·6H_2_O, 10 μg/L MnCl_2_, 10 μg/L (NH_4_)6Mo_7_O_24_·4H_2_O] and the cultures were shaken for 6 days (30 °C/200 rpm). After 6 days, mycelia were removed by centrifugation at 4000 rpm for 20 min, and 2 g of HP-20 resin (4%, w/v) was added to the supernatant. After an additional 12 h incubation (200 rpm), the resin was collected using cheese cloth, washed by 50 mL of H_2_O, and dried at room temperature for 20 min. The resins were then eluted with 15 mL of methanol for 2 h (200 rpm). The methanolic elution was concentrated in vacuo and dissolved in 500 μL of methanol. Each sample was centrifuged for 2 min to remove insoluble materials and then analyzed by UPLC-MS.

### Scaled Cultivation, Extraction, Isolation and Structure Determination of 1

*S. albus* J1074 containing Int_DFD0097_w371 was shaken in 15 individual 2 L flasks containing 400 mL of R5a medium for 6 days as described above. Then, 6 L of cultures were combined and mycelia were removed by centrifugation at 4000 rpm for 30 min. 240 g of Diaion HP-20 resin (4%, w/v) was added to the supernatant. After an additional 12 h incubation (200 rpm), the resin was collected with cheesecloth and washed with 2 L of H_2_O. The dried resin was eluted with 500 mL of methanol for 4 h (200 rpm) in a 2 L flask. The methanolic elution was concentrated in vacuo. Then, 250 mL methanol was added into the dried extract, C18 reversed phase silica gel was added, and the mixture was concentrated in vacuo. The C18-adsorbed extract was partitioned by medium-pressure liquid chromatography (100 g Gold HP C18 column, a linear gradient elution from 95% H_2_O/MeOH to MeOH for 20 min, 60 mL/min) and the fractions containing target peaks were combined. The combined fractions were dried under vacuum to yield **1** sufficiently pure for NMR spectroscopic characterization (174 mg). Then, the combined **1**-containing fractions were subjected to HPLC chromatography (XBridge Prep C18, 10 × 150 mm, 5 μm, 130 Å, 3.5 mL/min gradient elution from 50% to 80% CH_3_CN over 45 min, with 0.1% formic acid) to afford the pure form of **1** (140 mg). All NMR spectra of **1** were recorded at room temperature in CDCl_3_. ^1^H and ^13^C NMR data of **1** are presented in Table 1 and NMR spectra are located in SI Figures S5–12.

*Metamarin (***1***)*: white solid, [α]^24.6^_D_ = −60.9 (*c* 0.5, CH_3_OH); UV (CH_3_OH) λ_max_ 228, 254, 274, 298, 326, 362, 381, 405 nm; IR (film) *v*_max_ = 3341, 3310, 2961, 2939, 1642, 1544, 1455, 1410, 1031 cm^–1^ (SI Figure S4); ESI+ HRMS *m*/*z* 997.6102 [M+H–H_2_O]^+^ (calcd for C_55_H_81_N_8_O_9_^+^, 997.6121) (SI Figure S3).

### Marfey’s Method

Compound **1** (1.1 mg, 0.001 mmol) was dissolved in 1 mL of 6 N HCl (aq) and stirred at 100 °C for 2 h. The reaction mixture was dried in vacuo to afford the hydrolysate of **1**. Using identical conditions as for **1**, Fmoc-*N*-methyl-l-Leu (Chem-Impex; 1.0 mg, 0.003 mmol) and Fmoc-*N*-methyl-d-Leu (Alfa Aesar; 1.1 mg, 0.003 mmol) were separately hydrolyzed and dried in vacuo. The dried hydrolysates of **1**, Fmoc-*N*-methyl-l-Leu and Fmoc-*N*-methyl-d-Leu, in addition to l-Val (AMRESCO; 0.9 mg, 0.008 mmol) and d-Val (Sigma; 0.9 mg, 0.008 mmol), were separately suspended in 150 μL of deionized H_2_O to which 300 μL of *N*_α_-(2,4-dinitro-5-fluorophenyl)-l-alaninamide (L-FDAA; 10 mg/mL in acetone) and 70 μL of 1 M NaHCO_3_ (aq) were added. Each reaction mixture was heated at 37 °C for 2 h, dried in vacuo, resuspended in 500 μL of CH_3_OH and then diluted 10-fold for UPLC-HRMS analysis using the following chromatographic conditions: 5% to 95% B from 0.0 to 60.0 min, 95% B from 60.0 to 67.5 min, 95% to 5% B from 67.5 to 70.0 min, and 5% B from 70.0 to 75.0 min. Peaks corresponding to FDAA-derivatized amino acids were identified from extracted ion chromatograms for *m*/*z* 370.1357 ± 0.0025 ([M + H]^+^ of FDAA-Val) and 398.1670 ± 0.0025 ([M + H]^+^ of *N*-Me-l-Leu). Retention times for the FDAA-derivatized amino acid standards were as follows: l-Val (16.94 min), d-Val (13.76 min), *N*-Me-l-Leu (18.75 min) and *N*-Me-d-Leu (17.87 min). FDAA-l-Val and FDAA-*N*-Me-l-Leu were observed in the derivatized hydrolysate of **1** at retention times of 16.94 and 18.77 min, respectively.

### Antibacterial Assay against Nonmycobacteria

HPLC-purified **1** was used for all biological evaluation. Compound **1** was assayed in triplicate against eight bacterial strains and one yeast in 96-well microtiter plates using a broth microdilution method. For *Candida albicans*, *Enterococcus faecalis* and *Staphylococcus aureus*, overnight cultures were diluted 2000-, 1000-, and 10 000-fold in LB broth, respectively. For the other seven bacteria, *Acinetobacter baumannii*, *Bacillus subtilis*, *Escherichia coli*, *Enterobacter cloacae*, *Klebsiella pneumoniae*, *Micrococcus luteus*, and *Pseudomonas aeruginosa*, overnight cultures were diluted 5000-fold in LB broth. 100 μL of each culture dilution was added into 100 μL of LB broth containing **1** at 2-fold serial dilutions across a 96-well microtiter plate, and the final concentration of **1** ranged from 128 to 0.5 μg/mL. Rifampicin was included as the control. Then, the plate was statically incubated at 37 °C for 16 h. The lowest concentration of **1** that inhibited visible microbial growth was recorded as the miminum inhibition concentration (MIC).

### Antibacterial Assay Against *M. smegmatis* mc^2^ 155

*M. smegmatis* mc^2^ 155 was shaken in Middlebrook 7H9 broth (supplemented with 0.2% glucose, 0.2% glycerol, and 0.05% tyloxapol) for 48 h (37 °C/200 rpm). Then, the culture was diluted to an OD_600_ of 0.005, and 100 μL was added to 100 μL of 7H9 broth containing **1** at 2-fold serial dilutions across a 96-well plate, and the final concentration of **1** ranged from 128 to 0.5 μg/mL. Rifampicin was included as the control. The plates were statically incubated for 48 h at 37 °C and then 30 μL of Alamar Blue cell viability reagent (Thermo Fisher Scientific) was added. After an additional 24 h incubation, the wells that remained blue by visual inspection were deemed to contain inhibitory concentrations of **1**.

### Antibacterial Assay Against *M. tuberculosis*

*Mtb* H37Rv and multidrug-resistant strains (565, 7791, and TN800) were passaged in Middlebrook 7H9 broth (supplemented with oleic acid-albumin-dextrose-catalase, 0.2% glycerol and 0.02% tyloxapol) at 37 °C to OD_600_ of 0.5–0.7. Then, the culture was diluted to an OD_600_ of 0.005, and 100 μL of diluted cultures were distributed in 96-well plates. 100 μL of the **1** gradient dilutions were added to individual wells and the final test concentrations ranged from 5 to 0.009 μg/mL. Rifampicin was included as the control. The plates were incubated at 37 °C with room air oxygen and 5% CO_2_. After incubation for 6 days, 12.5 μL of 20% Tween 80 and 20 μL of Alamar Blue cell viability reagent were added, the cultures were incubated for another 24 h, and the absorbance was read at 570 nm and normalized to 600 nm per the manufacturer’s instructions.

### Murine Macrophage Infection

The activity of **1** against intracellular *Mtb* was determined by infecting J774A.1 mouse macrophages (Sigma-Aldrich) with the mc^2^ 6206 strain of *Mtb* harboring the mLux plasmid based on published protocols.^34,35^ Briefly, the macrophages were suspended in Dulbecco’s Modified Eagle Medium (DMEM, Sigma-Aldrich) supplemented with 10% Fetal Bovine Serum (FBS, Sigma-Aldrich) to a concentration of (4–5) × 10^5^ cells/mL. Flat bottom 96-well white plates were seeded with 100 μL of the macrophage suspension and incubated overnight to allow cells to adhere to the plates. The strain mc^2^ 6206 with the mLux plasmid was grown to mid log phase (OD_600_ = 0.5–0.7). Then, *Mtb* cultures were spun down, washed once in phosphate-buffered saline (PBS, Sigma-Aldrich), and resuspended in DMEM containing 10% FBS, pantothenic acid (50 μg/mL) and leucine (50 μg/mL). The assay plates were then inoculated with 100 μL of mc^2^ 6206 with the mLux plasmid at a multiplicity of infection of 1:10. The plates were incubated for 4 h at 37 °C and 5% CO_2_ to allow *Mtb* to infect the macrophages. Then, the infection was washed with 100 μL PBS, and 100 μL of DMEM containing 10% FBS, pantothenic acid and leucine was added and incubated for 1 h. The plates were washed twice with 100 μL of PBS. Then **1** with serial dilutions (from 64 to 0.0004 μg/mL) in 100 μL per well of DMEM containing 10% FBS, pantothenic acid and leucine were added to the plates at the desired concentrations. Rifampicin was used as the control. The plates were incubated at 37 °C for 72 h. Residual *Mtb* cell viability inside macrophages was determined by luminescence measurement on a Spark multimode microplate reader (Tecan). Dose response curves were generated by nonlinear regression in GraphPad Prism v8 and plotted as the logarithm of concentration vs normalized percent cell viability. The **1** concentration that caused inhibition of 50% cell viability (IC_50_) was determined from the dose–response curves. Each treatment was carried out in triplicate and the entire experiment was repeated twice.

### Overexpression and Purification of ClpC1

The *Mtb* ClpC1 ORF was obtained by PCR from the genomic DNA of *Mtb* H37Rv using the primers *Mtb*-ClpC1-F and *Mtb*-ClpC1-R. The PCR product was ligated into the expression vector pET28c between the *Nde* I and *Hind* III sites to generate pET28c-*clpC1*, which was verified by Sanger sequencing. ClpC1 overexpression and purification were performed as previously described.^36^ Briefly, the *E. coli* BL21(DE3) strains harboring the plasmid pET28c-*clpC1* were grown in 200 mL LB medium at 37 °C to an OD_600_ of 0.6–0.8. Isopropyl β-D-1-thiogalactopyranoside (IPTG) was added into the medium to induce the protein expression at 16 °C and the final concentration of IPTG was 1 mM. Then, the cells were harvested, disrupted and centrifuged to remove the debris. The supernatants were loaded on a Ni-NTA agarose column (GE healthcare). After washing the column by buffer A (20 mM Tris-HCl, 500 mM NaCl, 10% glycerol, pH 7.9) with gradient imidazole (20, 50, and 75 mM), the ClpC1 proteins bound to the beads were eluted with buffer B (20 mM Tris-HCl, 500 mM NaCl, 500 mM imidazole, 10% glycerol, pH 7.9). The elution buffer was exchanged with protein storage buffer (50 mM Tris-HCl, 50 mM KCl, 2 mM DTT, 10% glycerol, pH 7.9) using the PD-10 desalting column (GE healthcare). The purities of ClpC1 were detected by NuPAGE 4–12% Bis-Tris Protein Gel (Invitrogen), and the concentrations of ClpC1 were determined by the Quick Start Bradford Protein Assay Kit 2 (Biorad).

### Measurement of ClpC1 ATPase Activity

The ATPase activity of *Mtb* ClpC1 was determined by the BIOMOL Green-based protein phosphatase assay according to the colorimetric quantitation of released free phosphate (Enzo Life Sciences). The reaction assay was carried out in buffer (100 mM Tris, 200 mM KCl, 8 mM MgCl_2_, pH 7.5) and the total reaction volume was 50 μL. The final concentrations of ClpC1 and ATP were 1 μM and 100 μM, respectively. **1** dissolved in DMSO was added at 0.1, 1.0, and 10 μM as the final concentrations in each well of the Corning black 96-well plate with flat clear bottom. Then, the reaction mixture was incubated at 37 °C for 1 h and 50 μL of BIOMOL Green solution was added. After an additional 20 min incubation at room temperature, the OD_620_ of the reaction mixture was measured on an Infinite M Nano instrument (Tecan). The ATPase rate was calculated from the concentration of free phosphate released from ATP by ClpC1 in three independent experiments. The presence of DMSO did not affect the ClpC1 ATPase activity.

### Measurement of Proteolytic Activity of the ClpC1/P1/P2 Complex

The proteolytic activity of the ClpC1/P1/P2 complex was determined by degradation of the substrate fluorescein isothiocyanate (FITC)-casein (Sigma-Aldrich).^37^ The reaction assay was carried out in buffer (100 mM Tris, 200 mM KCl, 8 mM MgCl_2_, pH 7.5) and the total reaction volume was 100 μL. The final concentrations of ClpC1, ClpP1/P2, FITC-casein and ATP were 1 μM, 2 μM, 0.3 μM, and 2 mM, respectively. The proteins ClpP1 and ClpP2 were obtained from the *Mtb* ClpP Protease Assay Kit (ProFoldin). To measure ClpC1/P1/P2-mediated FITC-casein degradation activity in the presence of **1** dissolved in DMSO, **1** was added at 0.1, 1.0, and 10 μM in each well of the Corning black 96-well plate (flat clear bottom). The increase of FITC-casein fluorescence upon its degradation was monitored at 485 nm excitation and 535 nm emission in three independent experiments on an Infinite M Nano instrument (Tecan), and the initial fluorescence intensity was set to 100.

## Supporting Information Available

Supplemental tables, figures and 1D/2D NMR spectra and proposed biosynthesis of **1** (PDF)
