# Supplementary material for: Biosynthetic Interrogation of Soil Metagenomes Reveals Metamarin, an Uncommon Cyclomarin Congener with Activity against Mycobacterium tuberculosis
Source: J Nat Prod. 2021 Feb 23;84(4):1056–66. doi: 10.1021/acs.jnatprod.0c01104 (PMC8068612; doi:10.1021/acs.jnatprod.0c01104)
Supplement: Supplementary file 1 — np0c01104_si_001.pdf [file np0c01104_si_001.pdf]

# Supplemental Information for:

**Title: Biosynthetic interrogation of soil metagenomes reveals metamarin, an uncommon cyclomarin congener with activity against *Mycobacterium tuberculosis***

**Authors:** Lei Li[†], Logan W. Maclntyre[†], Thahmina Ali[†], Riccardo Russo[‡], Bimal Koirala[†], Yozen Hernandez[†], and Sean F. Brady[†,*]

**Author affiliation**:
[†] Laboratory of Genetically Encoded Small Molecules, The Rockefeller University, 1230 York Avenue, New York, NY 10065, United States.
[‡] Rutgers, The State University of New Jersey, International Center for Public Health, 225 Warren Street, Newark, NJ 07103, United States.

**Corresponding Author:** Sean F. Brady

**Contact**: Laboratory of Genetically Encoded Small Molecules
The Rockefeller University
1230 York Avenue
New York, NY 10065
**Phone:** 212-327-8280
**Fax:** 212-327-8281
**Email:** sbrady@rockefeller.edu

**Table of Contents:**

1. Table S1: List of PCR primers used in this study
2. Table S2: Antimicrobial activity of **1** against various microorganisms
3. Figure S1: Analysis of DFD1103_w205 gene cluster
4. Figure S2: Construction of the cosmid Int_DFD0097_w371
5. Figure S3: High-resolution mass spectrum and structure of **1**
6. Figure S4: Infrared (IR) spectrum of **1**
7. Figure S5: $^{1}$H NMR spectrum of **1** in $CDCl_3$ (600 MHz)
8. Figure S6: $^{13}$C NMR spectrum of **1** in $CDCl_3$ (600 MHz)
9. Figure S7: COSY NMR spectrum of **1** in $CDCl_3$ (600 MHz)
10. Figure S8: $^{1}$H-$^{13}$C HSQC NMR spectrum of **1** in $CDCl_3$ (600 MHz)
11. Figure S9: $^{1}$H-$^{13}$C HMBC NMR spectrum of **1** in $CDCl_3$ (600 MHz)
12. Figure S10: $^{1}$H-$^{15}$N HSQC spectra of **1** in $CDCl_3$ (500 MHz)
13. Figure S11: $^{1}$H-$^{15}$N HMBC spectra of **1** in $CDCl_3$ (500 MHz)
14. Figure S12: All COSY, $^{1}$H-$^{13}$C HMBC and $^{1}$H-$^{15}$N HMBC NMR correlations for **1**
15. Figure S13: The structures of cyclomarin A, M10709 and **1**
16. Figure S14: Proposed biosynthesis of **1**

**Table S1** List of PCR primers used in this study

| Primers | Sequences (5'-3') | Description |
|---|---|---|
| A3F<br>A7R | GCSTACSYSATSTACACSTCSGG<br>SASGTCVCCSGTSCGGTA | AD degenerate primers for screening eDNA collection and cosmid libraries |
| DFD0383_w617_F<br>DFD0383_w617_R | ACGCCGAAGGGCGTCGGCGTCA<br>AGCAGGTCGTCACCCAAGAG | Clone recovery of cosmid DFD0383_w617 |
| DFD0097_w188_F<br>DFD0097_w188_R | ACGCCGGCACGGTCAACCTG<br>TCCCCGACCGGACGCTCCACCT | Clone recovery of cosmid DFD0097_w188 |
| DFD1103_w205_F<br>DFD1103_w205_R | TGGCGACGACGATCCTCGA<br>GCCAGCGCTTCGCCGACGA | Clone recovery of cosmid DFD1103_w205 |
| DFD0097_w371_F<br>DFD0097_w371_R | AAGGGCGTCGCCGTCACCCACA<br>AGGGTGGCACCGGTGGTGAAC | Clone recovery of cosmid DFD0097_w371 |
| DFD1080_w495_F<br>DFD1080_w495_R | CAAAGGCGTCGCCGTGACCCA<br>TGGTGAACGCCGAGACCAGGT | Clone recovery of cosmid DFD1080_w495 |
| *Mtb*_ClpC1_F<br>*Mtb*_ClpC1_R | GGAATTCCATATGATGTTCGAACGATTTACCGACCGT<br>CCCAAGCTTCTAGCCGCCCGCGCTGTGCGCTCCAGCCTT | Expression of the protein *Mtb* ClpC1 |

**Table S2** Antimicrobial activity of **1** against various microorganisms

| Organism | Strain | Metamarin MIC (μg $mL^{-1}$) | Cyclomarin A MIC (μg $mL^{-1}$) | Rifampicin MIC (μg $mL^{-1}$) |
|---|---|---|---|---|
| | H37Rv | 0.16 | 0.08 | 0.04 |
| *Mycobacterium tuberculosis* | 565 | 0.08 | 0.08 | >10 |
| | 7791 | 0.63 | 0.31 | 0.04 |
| | TN800 | 0.63 | 0.31 | >10 |
| *Mycobacterium smegmatis* | $mc^2$ 155 | 16.0 | 2.0 | 1.0 |
| *Micrococcus luteus* | NRRL B-1018 | 8.0 | 4.0 | 0.016 |
| *Bacillus subtilis* | 168 1A1 | >128 | >128 | 0.031 |
| *Enterococcus faecium* | Com15 | >128 | >128 | 4.0 |
| *Staphylococcus aureus* | USA300 | >128 | >128 | 0.016 |
| *Escherichia coli* | DH5α | >128 | >128 | 4.0 |
| *Acinetobacter baumannii* | ATCC 17978 | >128 | >128 | 2.0 |
| *Pseudomonas aeruginosa* | PAO1 | >128 | >128 | 32.0 |
| *Klebsiella pneumoniae* | ATCC 10031 | >128 | >128 | 8.0 |
| *Candida albicans* | SFB | >128 | >128 | 0.016 |

**a**

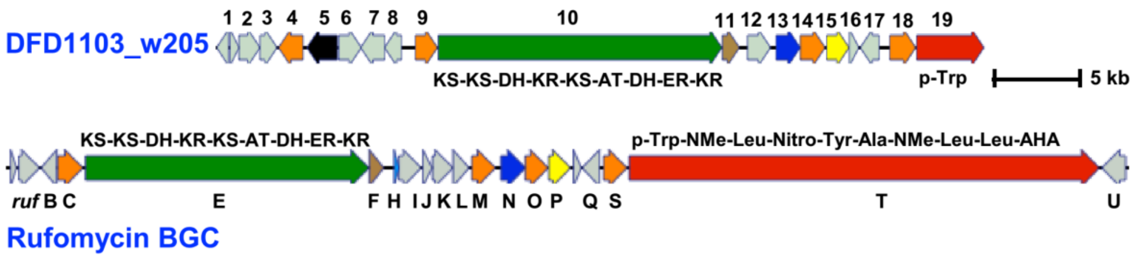


**b**

| UT60_w205_Gene | Rufomycin_Gene | Protein family | Proposed function | ID% |
|---|---|---|---|---|
| 1 | *rufJ* | DNA-binding protein | Unknown | 50% |
| 2 | *rufK* | ABC-type transporter | Transport | 70% |
| 3 | *rufL* | ABC-type transporter | Transport | 64% |
| 4 | *rufM* | P450 | COOH installation of AD05-Leu | 66% |
| 5 | | ClpX | Possible resistance gene | NA |
| 6 | | TetR transcriptional regulator | Regulation | NA |
| 7 | *rufU* | Lactate dehydrogenase | Unknown | 69% |
| 8 | *rufB* | *α*/*β*-Hydrolase | Unknown | 61% |
| 9 | *rufC* | P450 | AHA biosynthesis | 63% |
| 10 | *rufE* | Type I polyketide synthase | AHA biosynthesis | 52% |
| 11 | *rufF* | Thioesterase | AHA biosynthesis | 49% |
| 12 | *rufI* | Aminotransferase | AHA biosynthesis | 60% |
| 13 | *rufN* | Nitro oxide synthase | 3-Nitro-Tyr biosynthesis | 67% |
| 14 | *rufO* | P450 | 3-Nitro-Tyr biosynthesis | 70% |
| 15 | *rufP* | Indole PTase | *N*-prenyltransferase | 56% |
| 16 | *rufQ* | Aminotransferase subunit A | Unknown | 46% |
| 17 | *rufR* | Reductase | Unknown | 59% |
| 18 | *rufS* | P450 | p-Trp epoxide group biosynthesis | 62% |
| 19 | *rufT* | NRPS | Rufomycin peptide synthetase | 57% |

**Figure S1.** **a**) DFD1103_w205 and rufomycin gene clusters. **b**) Predicted functions of proteins encoded by DFD1103_w205 gene cluster. ID% represent amino acid identities of protein homologs encoded by the DFD1103_w205 and rufomycin gene clusters. pTrp and AHA represent *N*-(1,1-dimethyl-1-allyl)Trp and 2-amino-4-hexenoic acid, respectively.

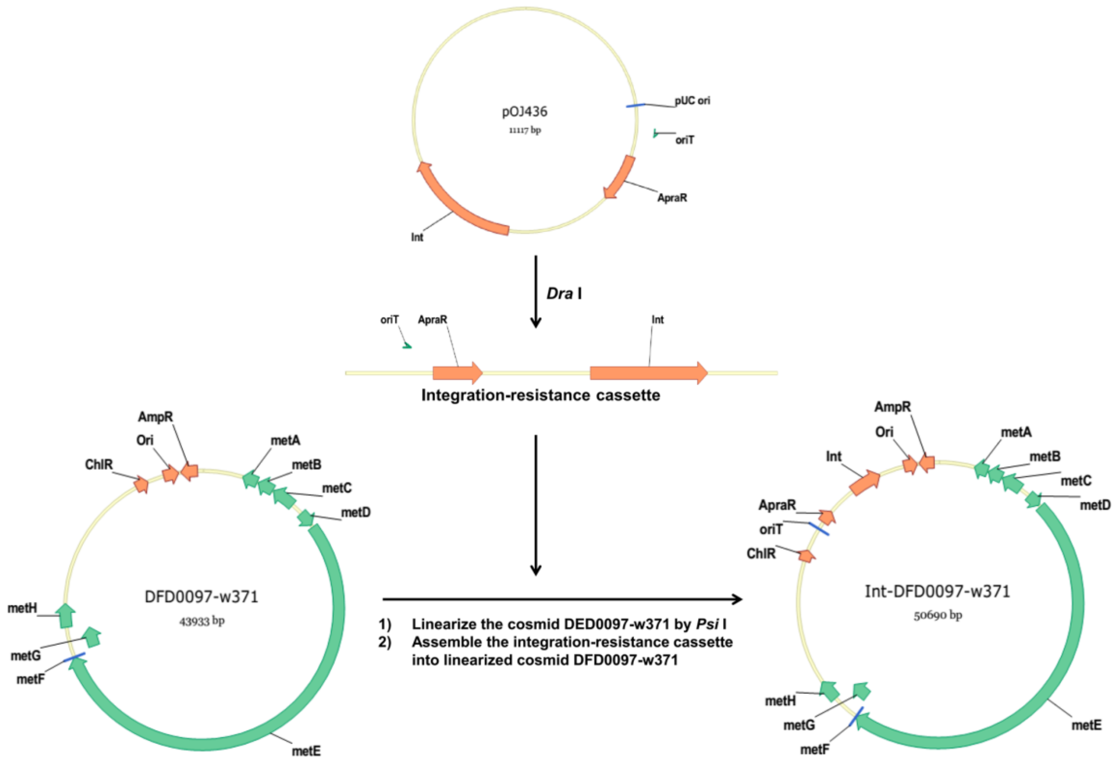


**Figure S2.** Construction of the cosmid Int-DFD0097-w371 that contains the entire BGC of **1**.

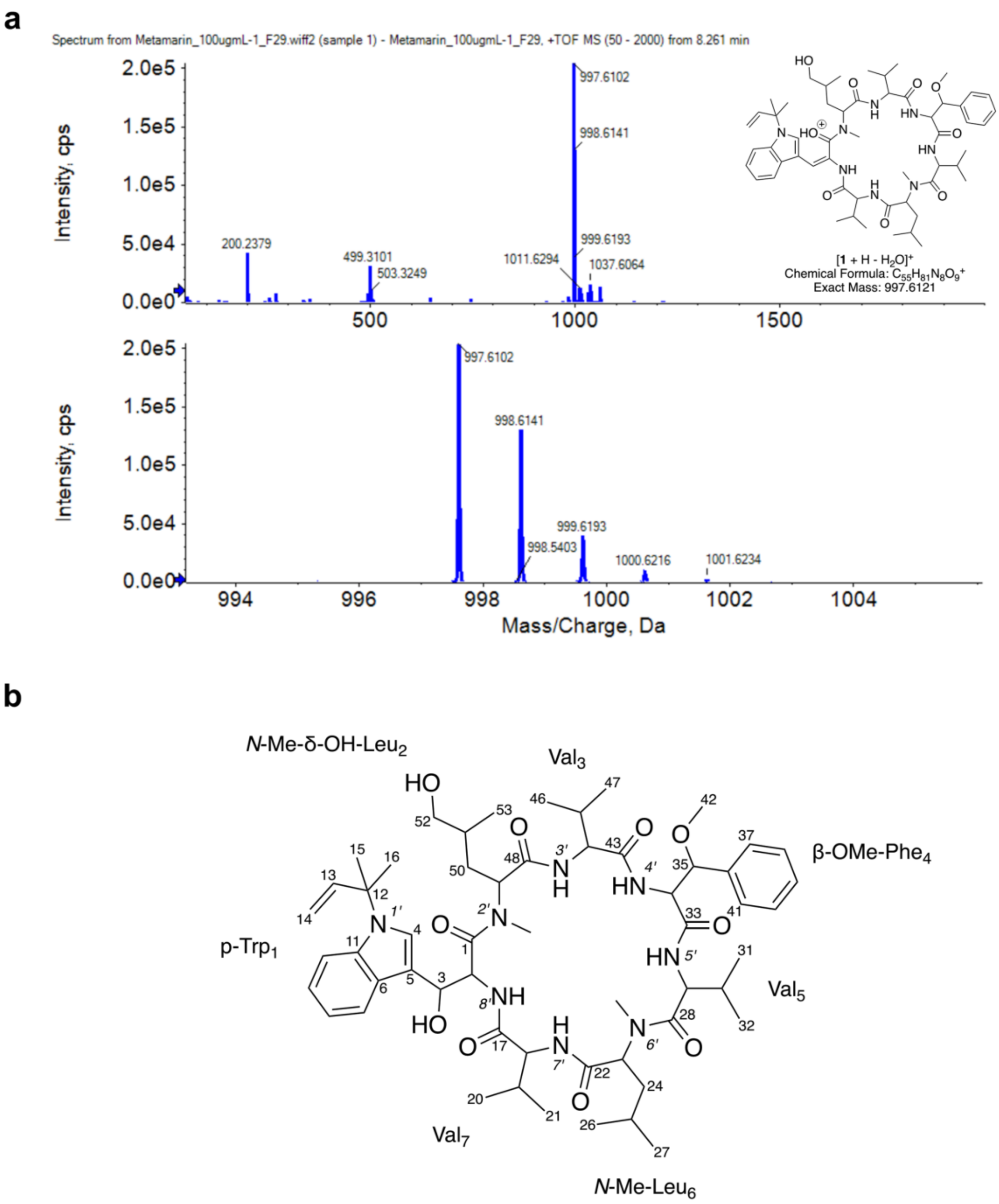


**Figure S3. a**) High-resolution mass spectrum of **1**. **b**) Structure of **1**.

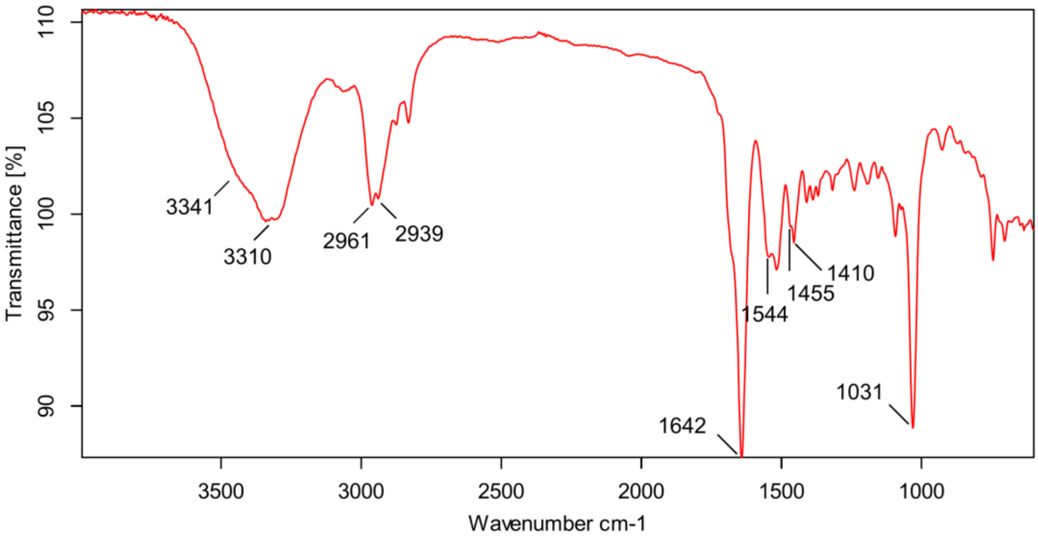

**Figure S4.** Infrared (IR) spectrum of **1**

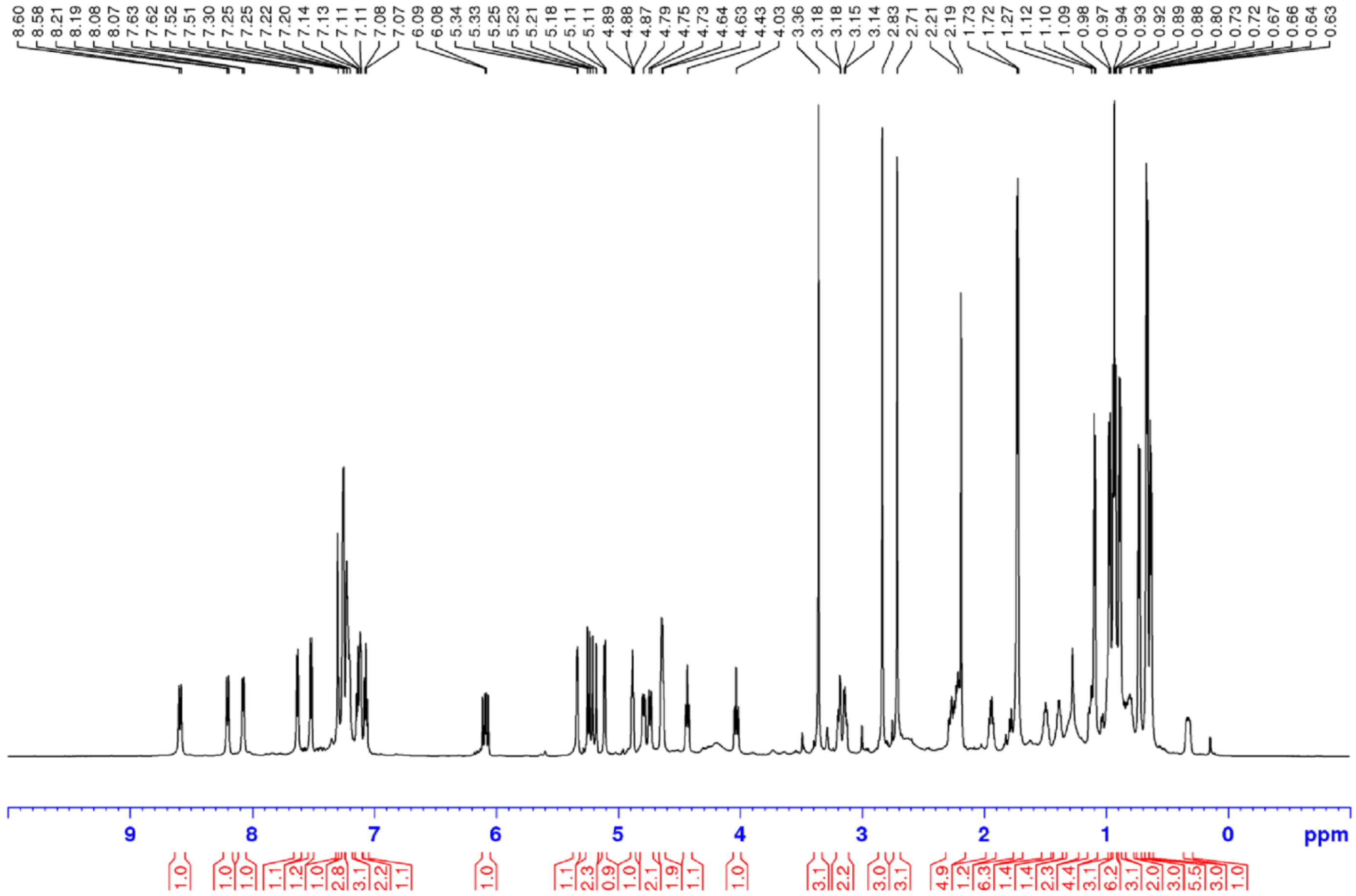

**Figure S5.** $^{1}H$ NMR spectrum of **1** in $CDCl_3$ (600 MHz)

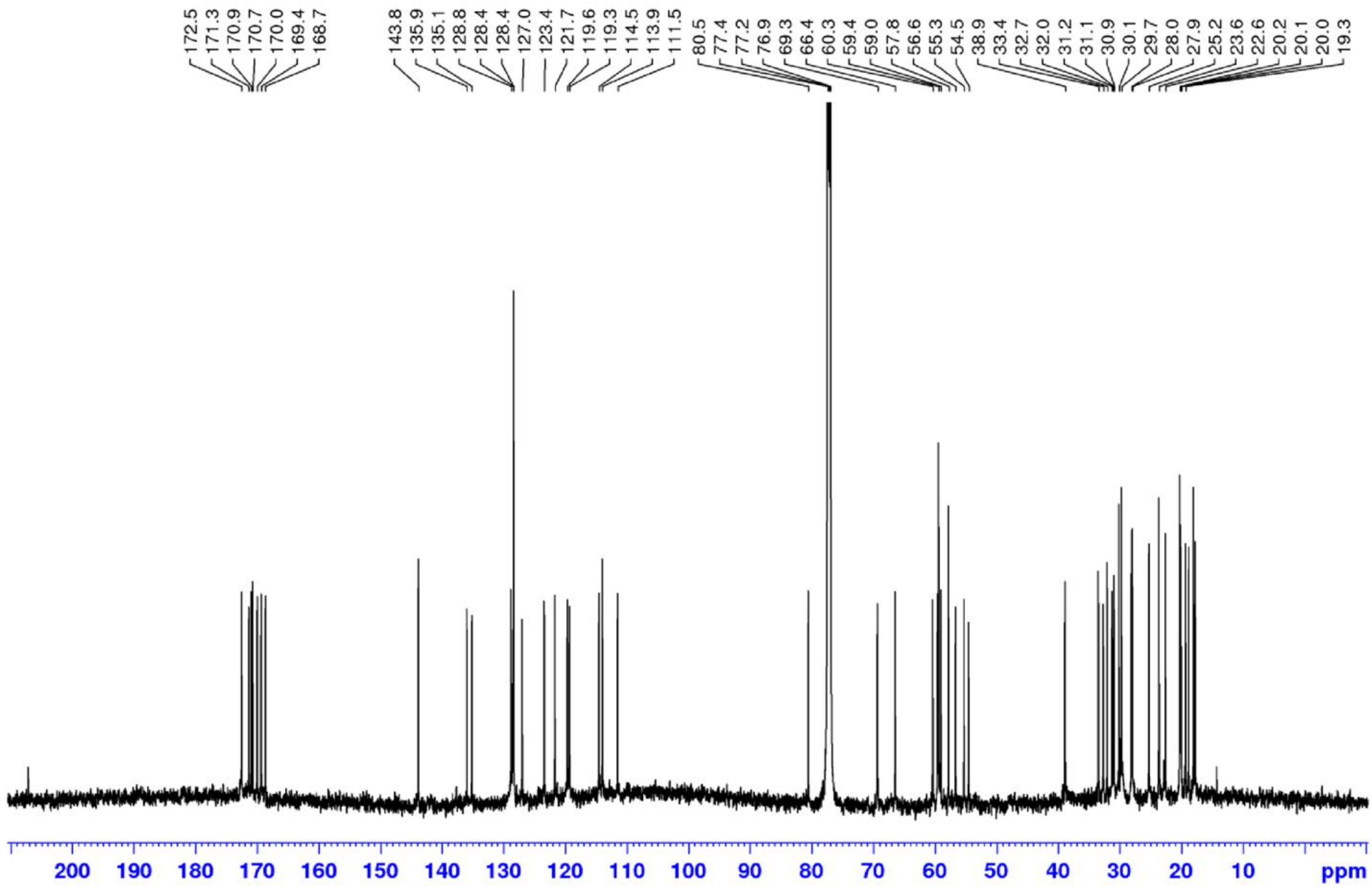


**Figure S6.** $^{13}C$ NMR spectrum of **1** in $CDCl_3$ (150 MHz)

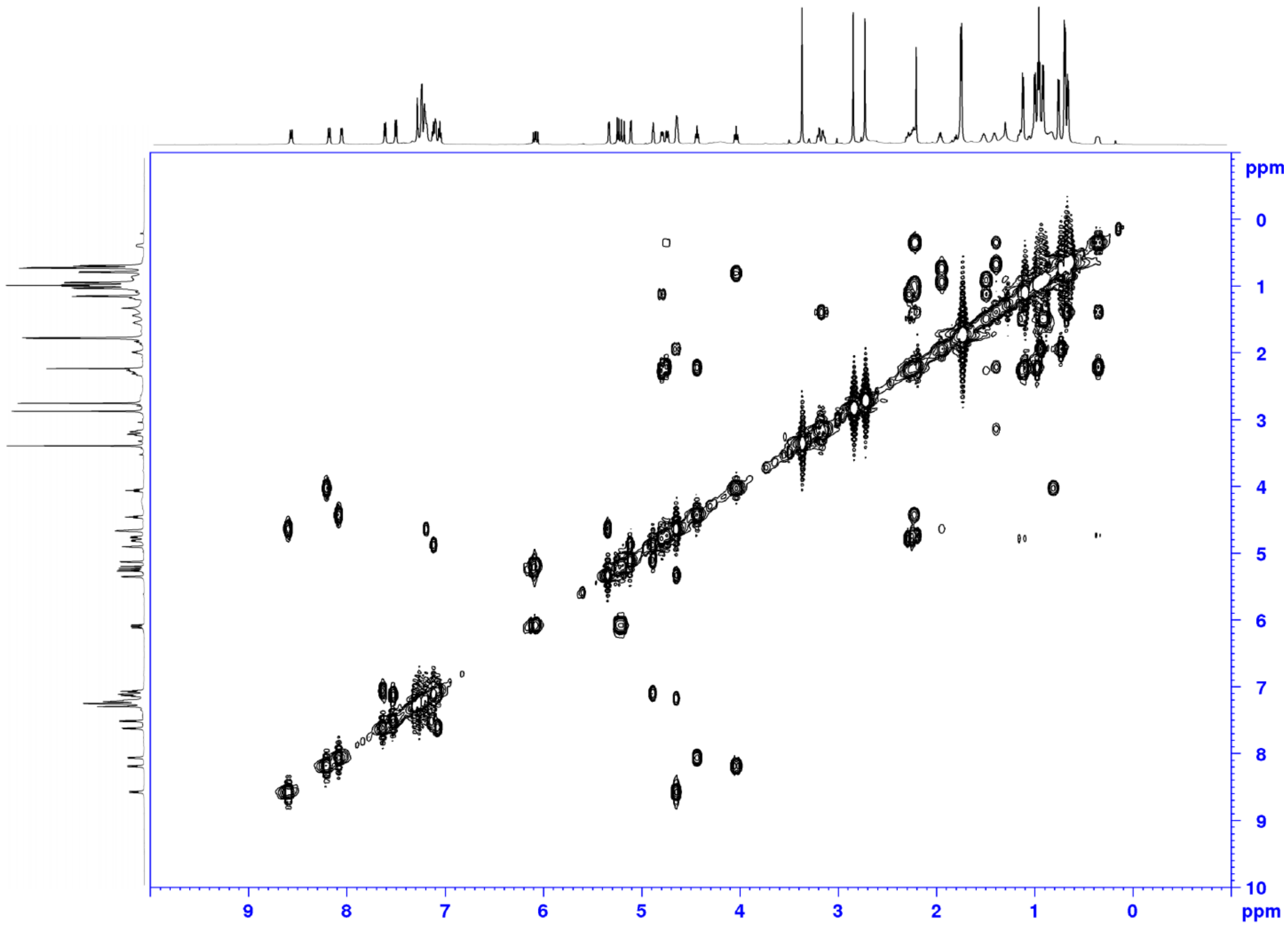


**Figure S7.** COSY NMR spectrum of **1** in $CDCl_3$ (600 MHz)

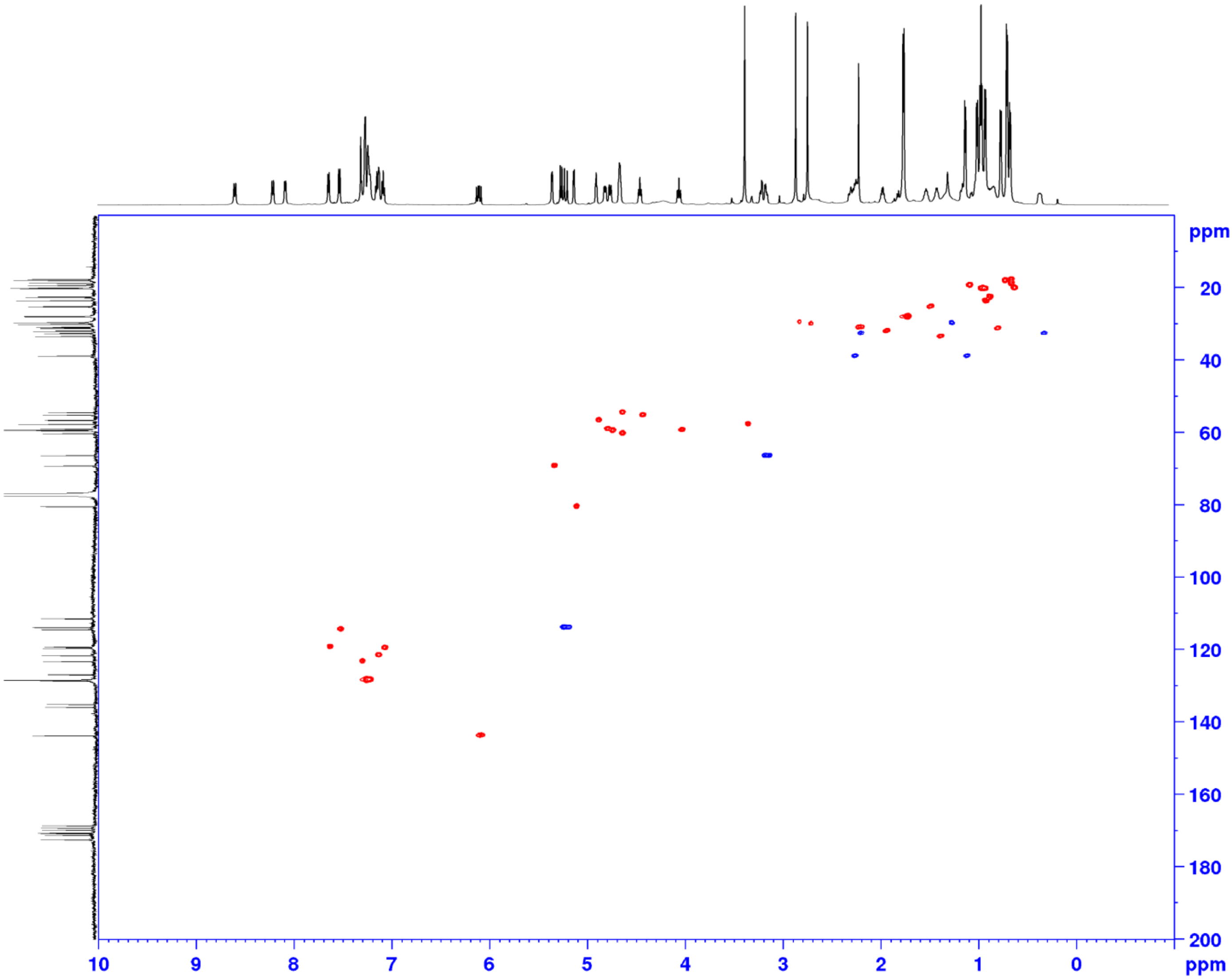


**Figure S8.** $^{1}H$-$^{13}C$ HSQC NMR spectrum of **1** in $CDCl_3$ (600 MHz)

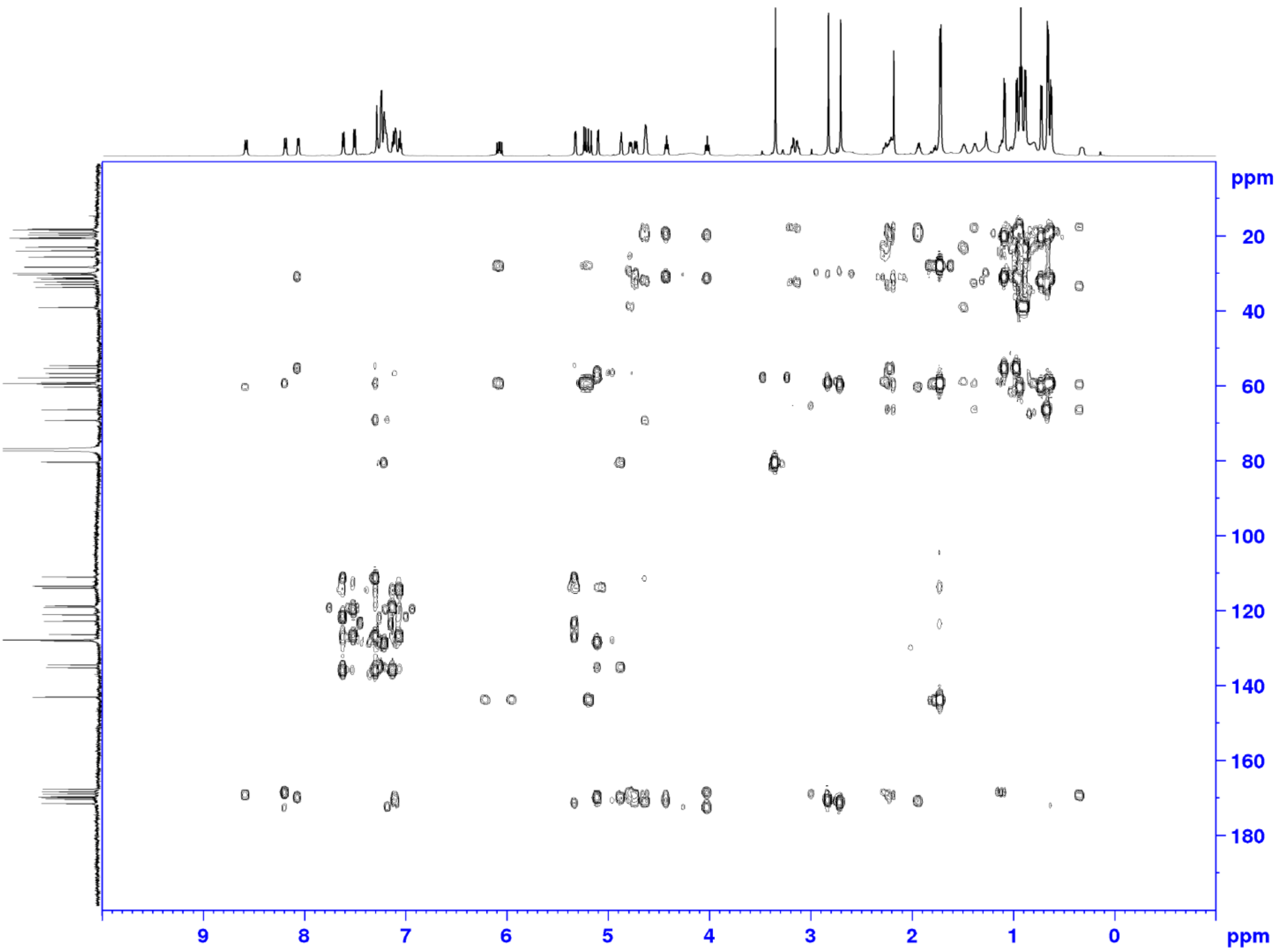


**Figure S9.** $^{1}H$-$^{13}C$ HMBC NMR spectrum of **1** in $CDCl_3$ (600 MHz)

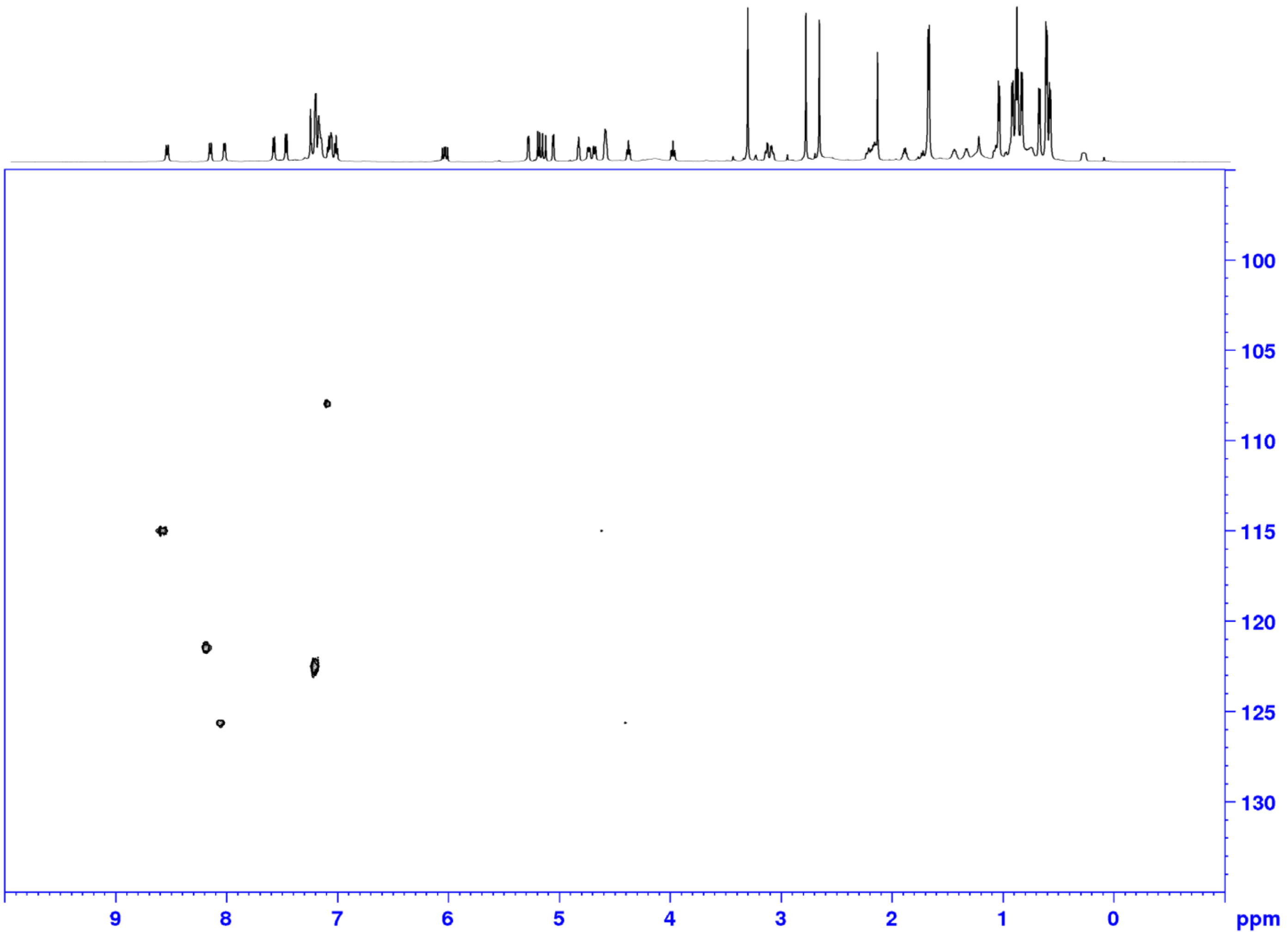


**Figure S10.** $^{1}H$-$^{15}N$ HSQC spectrum of **1** in $CDCl_3$ (500 MHz)

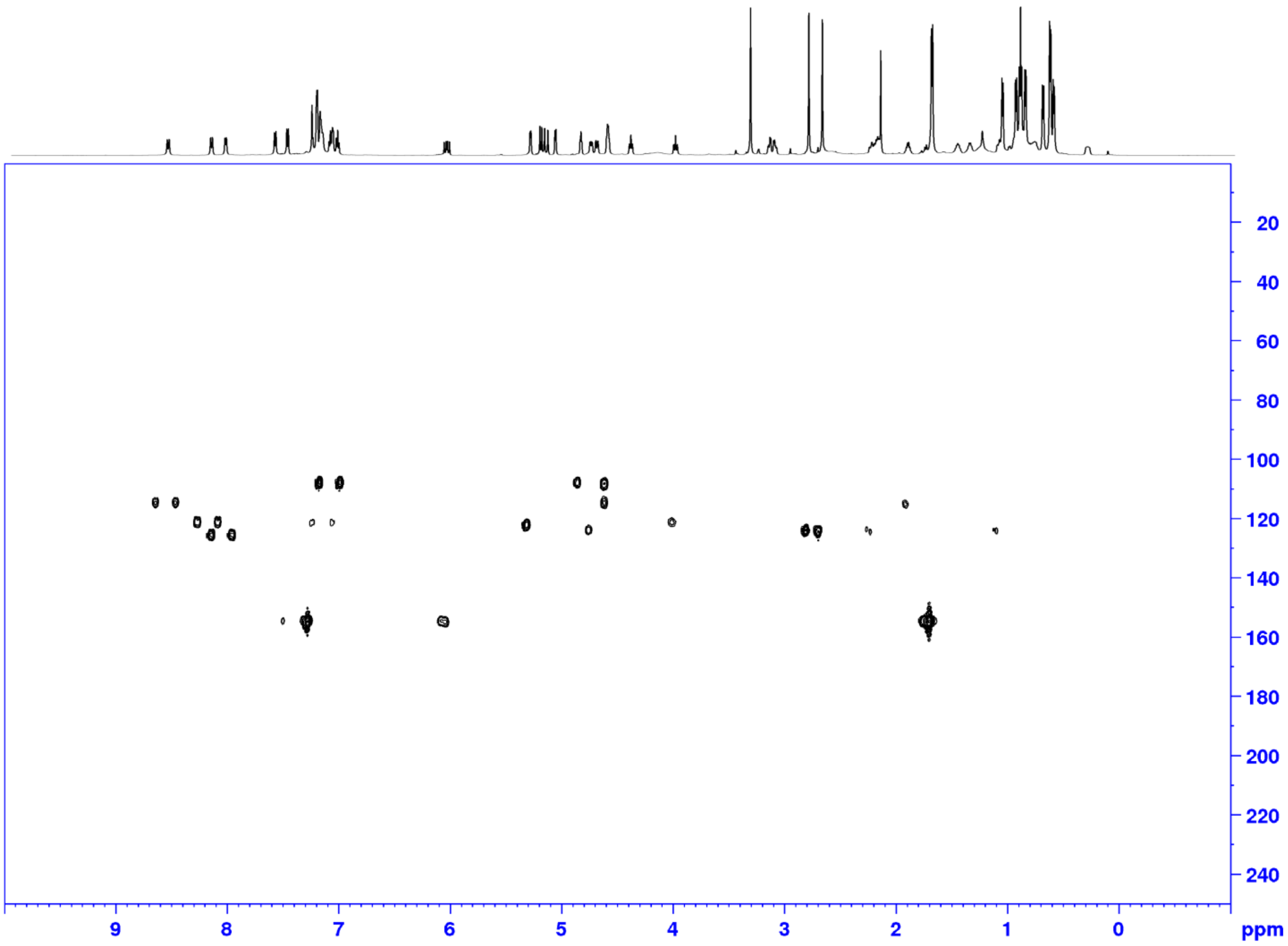


**Figure S11.** $^{1}H$-$^{15}N$ HMBC spectrum of **1** in $CDCl_3$ (500 MHz)

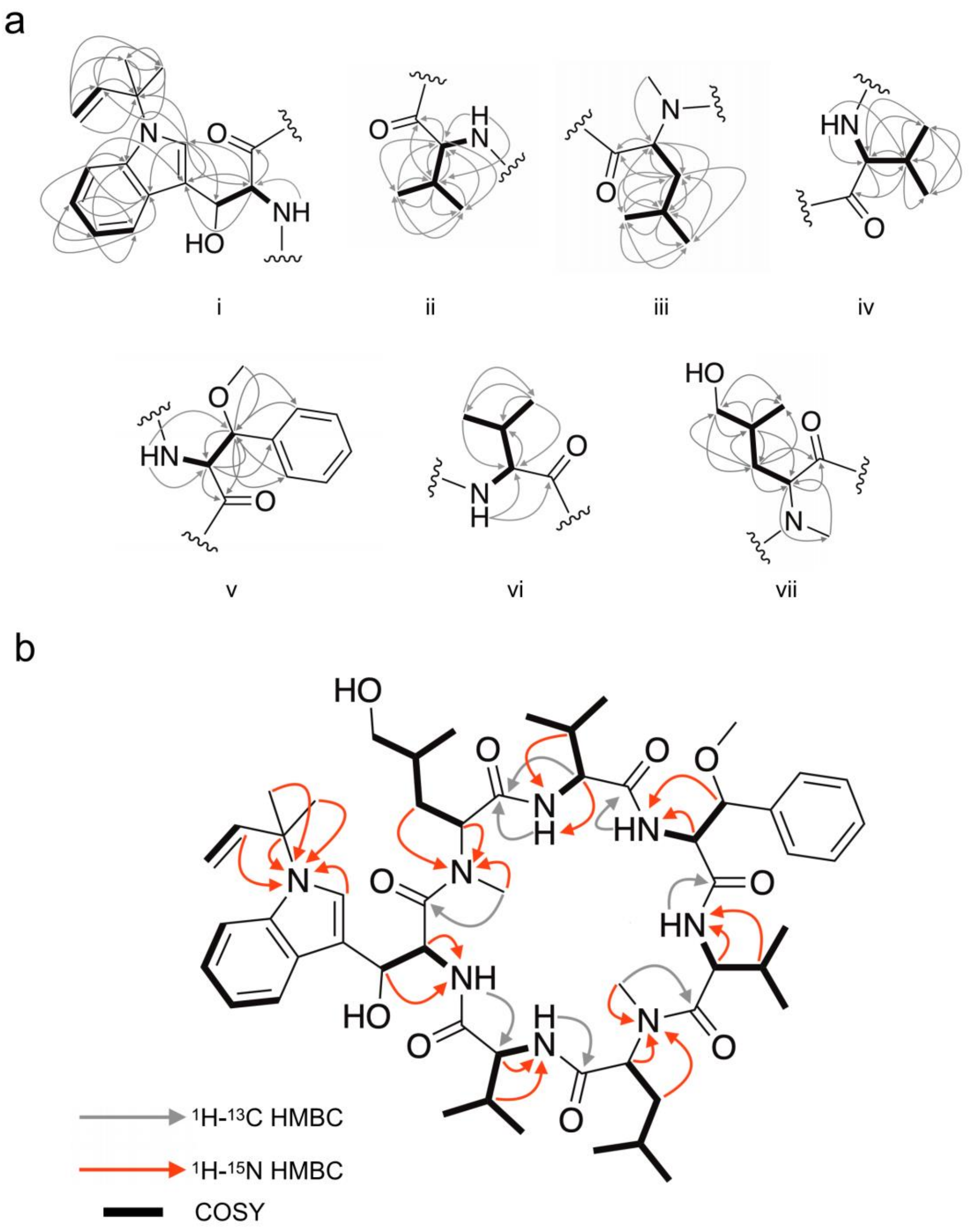


**Figure S12.** All COSY, $^{1}H$-$^{13}C$ HMBC and $^{1}H$-$^{15}N$ HMBC NMR correlations for **1**. **a**) Partial structures (i)-(vii) representing the constituent amino acid residues in **1** are depicted with all COSY and $^{1}H$-$^{13}C$ HMBC NMR correlations observed within each. (i) *N*-(1,1-dimethyl-1-allyl)-$\beta$-OH-$Trp_1$, (ii) *N*-Me-$\delta$-OH-$Leu_2$, (iii) $Val_3$, (iv) $\beta$-OMe-$Phe_4$, (v) $Val_5$, (vi) *N*-Me-$Leu_6$ and (vii) $Val_7$. **b**) $^{1}H$-$^{13}C$ HMBC NMR correlations that establish the connectivity of (i) -(vii) in addition to all $^{1}H$-$^{15}N$ HMBC correlations observed.

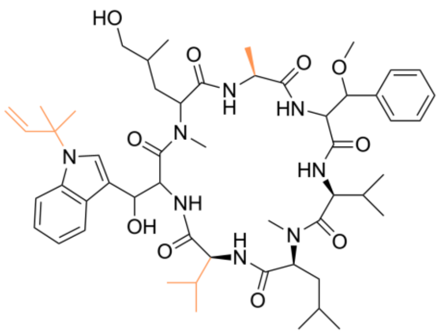

**Cyclomarin A**

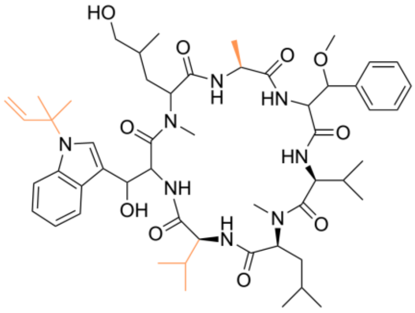

**M10709**

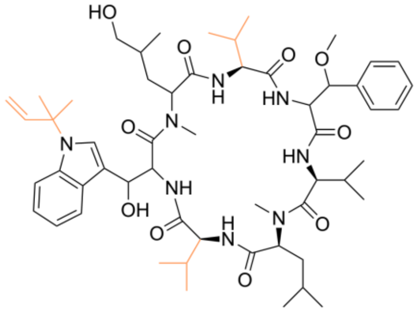

**1**

**Figure S13.** The structures of Cyclomarin A, M10709 and **1**

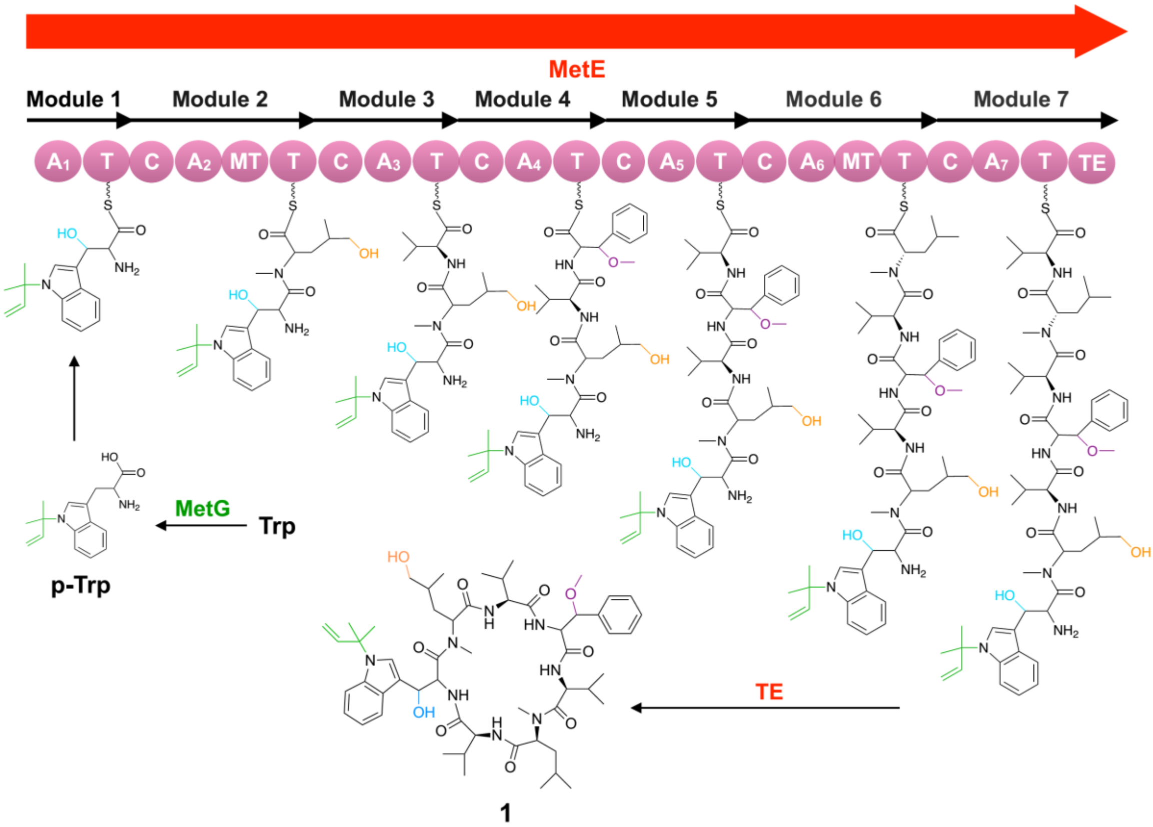


**Figure S14.** Proposed biosynthesis of **1**. Biosynthesis of **1** is predicted to follow a colinear extension model of modular NRPS system. C, condensation domain; A, adenylation domain; T, thiolation domain; TE, thioesterase domain; MT, *N*-methyltransferase domain. p-Trp represents *N*-(1,1-dimethyl-1-allyl)Trp.
